# Highly polygenic architecture of antidepressant treatment response: Comparative analysis of SSRI and NRI treatment in an animal model of depression

**DOI:** 10.1002/ajmg.b.32494

**Published:** 2016-10-01

**Authors:** Karim Malki, Maria Grazia Tosto, Héctor Mouriño‐Talín, Sabela Rodríguez‐Lorenzo, Oliver Pain, Irfan Jumhaboy, Tina Liu, Panos Parpas, Stuart Newman, Artem Malykh, Lucia Carboni, Rudolf Uher, Peter McGuffin, Leonard C. Schalkwyk, Kevin Bryson, Mark Herbster

**Affiliations:** ^1^ King's College LondonMRC SocialGenetic and Developmental Psychiatry Centre at the Institute of PsychiatryPsychology and Neuroscience (IOPPN)LondonUnited Kingdom; ^2^ LCIBGTomsk State UniversityTomskRussia; ^3^ Department of Computer ScienceUCLLondonUnited Kingdom; ^4^ Division of MedicineUCLLondonUnited Kingdom; ^5^ BirkbeckUniversity of LondonUnited Kingdom; ^6^ London School of Hygiene & Tropical MedicineUnited Kingdom; ^7^ Department of Computer Science Imperial College LondonUnited Kingdom; ^8^ Department of Pharmacy and BiotechnologyAlma Mater Studiorum University of BolognaBolognaItaly; ^9^ Department of PsychiatryDalhousie UniversityHalifaxNova ScotiaCanada; ^10^ School of Biological SciencesUniversity of EssexColchesterUnited Kingdom

**Keywords:** machine learning, SVM, transcriptomics, antidepressants, SSRI

## Abstract

Response to antidepressant (AD) treatment may be a more polygenic trait than previously hypothesized, with many genetic variants interacting in yet unclear ways. In this study we used methods that can automatically learn to detect patterns of statistical regularity from a sparsely distributed signal across hippocampal transcriptome measurements in a large‐scale animal pharmacogenomic study to uncover genomic variations associated with AD. The study used four inbred mouse strains of both sexes, two drug treatments, and a control group (escitalopram, nortriptyline, and saline). Multi‐class and binary classification using Machine Learning (ML) and regularization algorithms using iterative and univariate feature selection methods, including InfoGain, mRMR, ANOVA, and Chi Square, were used to uncover genomic markers associated with AD response. Relevant genes were selected based on Jaccard distance and carried forward for gene‐network analysis. Linear association methods uncovered only one gene associated with drug treatment response. The implementation of ML algorithms, together with feature reduction methods, revealed a set of 204 genes associated with SSRI and 241 genes associated with NRI response. Although only 10% of genes overlapped across the two drugs, network analysis shows that both drugs modulated the *CREB* pathway, through different molecular mechanisms. Through careful implementation and optimisations, the algorithms detected a weak signal used to predict whether an animal was treated with nortriptyline (77%) or escitalopram (67%) on an independent testing set. The results from this study indicate that the molecular signature of AD treatment may include a much broader range of genomic markers than previously hypothesized, suggesting that response to medication may be as complex as the pathology. The search for biomarkers of antidepressant treatment response could therefore consider a higher number of genetic markers and their interactions. Through predominately different molecular targets and mechanisms of action, the two drugs modulate the same *Creb1* pathway which plays a key role in neurotrophic responses and in inflammatory processes. © 2016 The Authors. *American Journal of Medical Genetics Part B: Neuropsychiatric Genetics* Published by Wiley Periodicals, Inc.

## INTRODUCTION

Major depressive disorder (MDD) is a pervasive psychiatric disorder characterized by a number of clinical symptoms including: persistent low mood, anhedonia, insomnia, low energy, feelings of guilt, and ideation of death or suicide. MDD is also associated with a range of social impairments, including educational and occupational problems and with an increased risk of developing systemic disease, such as cardiovascular disease and Type 2 diabetes [Mezuk et al., 2008; Goldstein et al., 2015]. Epidemiological studies have shown links between MDD and increased levels of mortality, due to either suicide or resulting diseases [Ferrari et al., 2013; Mullins et al., 2014]. In the US, MDD was reported to have a lifetime prevalence of 16.2% and comorbidity with at least one other DSM‐IV (diagnostic statistical manual IV) disorder of 72.1%, representing a major public health concern [Kessler et al., 2003].

Although quantitative genetic studies report moderate heritability estimates (between 40% and 50%), progress in uncovering the molecular substrate underpinning MDD has been slow [Levinson, 2006]. Highly powered GWAS (Genome‐Wide Association Study) have been less successful in uncovering common genetic variation associated with MDD than with other Axis‐I psychiatric disorders such as Schizophrenia [Schizophrenia Psychiatric Genome‐Wide Association Study (GWAS) Consortium, 2011]. To date, the largest published mega‐GWAS on MDD yielded negative findings [Major Depressive DisorderWorking Group of the Psychiatric Consortium et al., 2013]. Although GWAS hits for MDD have been recently announced, they still explain only a fraction of the variation in the disorder [Hyde et al., 2016]. One possible explanation for the difficulty in uncovering molecular variants associated with the pathology is that MDD is a highly heterogeneous disorder and selection of cases for GWAS studies still relies on clinical algorithms centered on symptom persistence and count that may be independent of any aetiological considerations [Malki et al., 2014]. This could point at multiple subtypes of depressive disorder and to genetic overlap between cases and controls. A second explanation is that depression is a complex, highly polygenic trait with a number of genes and environmental factors interacting in yet unclear ways [Uher, 2014]. It is likely that in isolation, no common variant of small penetrance can significantly account for the disorder. Traditional methods of analysis for Genome‐Wide data typically rely on a linear or logistic regression with an additive model that explores the association between single markers and a binary outcome. However, this loci‐by‐loci association method is poorly suited to capture gene–gene interaction effects (epistasis) or to consider the effects of clusters of genes across wide genomic distances.

The need to find fast and effective treatment for the pathology even in the absence of known aetiology remains a priority. To date there are over 30 pharmacological agents that have been shown to be effective for the treatment of MDD in clinical trials but less than 50% of patients respond favorably to the first prescribed drug [Thase et al., 2001]. It has long been hypothesized that response to drug treatment may be a less polygenic trait than the pathology itself. However, candidate gene studies informed by the known pharmacodynamic and pharmacokinetic mode of action of several classes of antidepressant drugs have yet to uncover a reliable genetic biomarker with clinical significance [Uher et al., 2012]. To date, the largest Genome‐Wide Pharmacogenetic Association Study on antidepressant treatment response has yielded negative results, suggesting that response to pharmacotherapy may itself be a complex, highly polygenic trait [Tansey et al., 2012].

Complex traits, including Axis‐I psychiatric disorders, have a multidimensional genetic architecture consisting of many small effect‐size variants, which only together through regulatory, stochastic events, gene–gene (epistasis) and gene‐environment interactions can account for individual differences in a clinically meaningful way. As a result, identification of groups of genes showing a moderate change in expression, but together significantly associated with the trait of interest, is likely to provide a powerful insight into the underlying molecular mechanisms by highlighting a mutual function [Mootha et al., 2003; Subramanian et al., 2005]. Multivariate methods of analysis offer advantages as identification of individual genes associated with complex traits is often difficult to interpret from a biological stand‐point and results are often poorly reproduced [Frantz, 2005].

Supervised machine learning approaches are a powerful tool for the classification of multidimensional data. Several methods in statistical learning use a multivariate approach to the entire dataset and are capable of considering interactions [Iniesta et al., 2016]. Several optimization techniques, including kernel functions and model parameters, allow the algorithms to detect patterns of statistical regularity across a sparsely distributed signal and use this information to make meaningful classifications and predictions. Application of statistical learning approaches to Psychiatry is more common in the classification of blood cell transcriptomic data [Marquand et al., 2008; Schwarz et al., 2010]. Although these studies point at the possibility of improving diagnosis of psychiatric disorders by successfully identifying trait‐specific gene expression signatures, they have not been widely used to uncover etiological mechanisms. Reports of these methods successfully dissecting complex psychiatric disorders strongly implies that the application of these methods to transcriptomic data, derived from disorder‐relevant brain tissue, could provide insight into underlying molecular and neurobiological mechanisms.

In this study we have evaluated, optimized and applied a range of machine learning algorithms to a mouse pharmacogenomic study from the GENDEP project [Malki et al., 2011]. By using this approach, we aim to identify groups of genes, and therefore biological pathways, underlying the differential response to two of the most popular classes of antidepressant drugs: Selective serotonin reuptake inhibitors (SSRIs) and norepinephrine reuptake inhibitor (NRIs).

## MATERIALS AND METHODS

### Design

The Genome‐based Therapeutic Drugs for Depression (GENDEP) is a large‐scale, multidisciplinary, multicenter study that focuses on the prediction of therapeutic responses and adverse effects to AD and on the molecular aetiology of Major Depressive Disorder (MDD) [Uher et al., 2010]. In this study we used hippocampal gene expression from an animal model of depression in a discovery‐replication design. The hippocampus was chosen at the time of the study because it is a brain region that can be dissected consistently in mouse and has been implicated in the regulation of mood in human studies [Santarelli et al., 2003; Surget et al., 2011]. Hippocampal neurogenesis has been repeatedly demonstrated as an important process for the behavioural effects of antidepressants in both rodents and non‐human primates [Perera et al., 2011]. The study used four strains of well‐characterized inbred mice (129S1/SvImJ, C57LB/6J, DBA/2J, and FVB/NJ), two depressogenic protocols and a control condition (maternal separation, chronic mild stress and control), two sexes, two drug administration regimes (chronic and acute) and two drug treatments (nortriptyline, escitalopram, or saline). This yielded a balanced design with 144 experimental cells. Supervised and non‐supervised machine‐learning and regularization methods, training and testing on independent samples were applied using probesets as features and drug (active drug or control) as outcome classes. The high‐dimensional structure data were first explored in a multi‐class design (three classes; two drugs, and one control condition) and then using a simpler binary classification, which compared each of the two drugs individually against a control (saline) condition. Probe‐sets were treated as feature variables and each of the animals as instances that can be represented as data points in a 37,231 (number of probes) dimensional feature space. Together with the corresponding factor (response) labels, supervised learning and penalized regularization approaches have been used to classify drug labels into their respective classes. Feature reduction methods were then used to extract those feature sets with higher probability of association with outcome class.

### Animals

A total of 144 male and female mice from the following four strains were used in this study in a balanced design: 129S1/SvImJ (129), C57LB/6J (C57), DBA/2J (DBA), and FVB/NJ (FVB). Each mouse represents an experimental group, which differs by combinations of strain, sex, stress, AD, and AD administration schedule. Animals used for this expression study were not behaviorally tested. However, a parallel large‐scale behavioral study with a matching design was previously conducted on a different set of animals [Binder et al., 2011]. All animals were bred in the barrier unit at the Institute of Psychiatry, London, UK. All housing and experimental procedures were carried out in accordance with the UK Home Office Animals (Scientific Procedures) Act 1986.

### Antidepressant Drugs

The GENDEP project used the same two‐antidepressant drugs across all of its treatment arms: the NRI nortriptyline (4 mg/kg) and escitalopram (5 mg/kg) representing the most two common classes of AD's. Clinical trials have shown that both classes of drugs are effective for the treatment of MDD although they have distinct modes of action [Sánchez and Hyttel, 1999; Sanchez et al., 2003]. SSRIs have no effect on noradrenaline reuptake while NRIs have up to 100 times higher affinity for the noradrenaline compared to the serotonin neurotransmitter.

### Dosage Pilot Study

A pilot study was conducted to determine a drug dosage that would elicit a behavioral response in the absence of an effect of locomotion or anxiety. Based on the pilot study, a dose of 5 mg/kg of escitalopram and 4 mg/kg of nortriptyline was chosen. Two drug administration regimes were explored (chronic‐14 days and acute 24 hr). All animals not in the chronic condition were injected with saline for 14 days to balance any stress from the intraperitoneal injections. Linear models did not reveal an effect of drug administration regime. Further information on the pilot study is available elsewhere [Binder et al., 2011].

### mRNA Extraction and Gene Expression Profiling

Brains were dissected and hippocampi frozen on dry ice. Total RNA was extracted from frozen hippocampal tissue and 3‐ug RNA was processed using the One Cycle Target Labelling kit (Affymetrix, Santa Clara, CA) and hybridized to the mouse MOE430v2 Gene Expression Array (Affymetrix) following standard Affymetrix protocols. At the time of the experiment, the Affymetrix Mouse MOE430v2 provided the most comprehensive coverage of the mouse genome. These arrays contain multiple probe pairs for each of the 45,101 probe sets, providing several independent measures for 39,000 transcripts corresponding to over 19,000 well characterized mouse genes.

### Statistical Analysis

Data from 144 Affymetrix mouse whole‐genome oligonucleotide arrays (MOE430v2) was normalized and summarized into probe sets by Robust Multichip Average (RMA) method [Irizarry et al., 2003]. Quality control of the resulting scans, including raw intensity distributions, profile correlations, and multivariate analysis indicated inconsistent quality with ten arrays, which were removed and QC analysis repeated. As part of the QC procedure, r and post‐normalisation boxplots of probe log‐intensity distributions were plotted to compare median intensities across arrays. These showed near identical distribution patterns following RMA normalization. Density plots of log‐intensity distribution of each array were subsequently superimposed to check for any deviation between‐arrays. Identification of intensity‐dependent biases was performed by visual inspection of MA plots, which are derived by computing pairwise‐comparison of log‐intensity of each array to a reference array. The Relative Log Expression (RLE) values were then computed by calculating the ratio between the expression of a probe‐set and the median expression of this probe‐set for each probe sets across all arrays. The results show a ratio of around 0 on a log‐scale, which is expected as most probe‐sets should remain invariant across the array. Percentage of present calls plots using Affymetrix MAS 5.0 show good consistency across arrays. Positive and negative controls distributions were plotted to check for non‐uniform hybridization or gridding problems, both showing clustering around a (0,0) center. RNA quality control was conducted using 3′/5′ ratio for beta‐actin and GAPDH. Values were well within commonly used threshold of three for beta‐actin and 1.25 for GAPDH showing consistently good quality. RNA degradation plots were also visually inspected to explore the directionality of degradation from the 5′ to the 3′ end and for consistency in the rate (slope) of degradation. Lastly, we used, heatmaps, PCA and hierarchical clustering to uncover potential outliers but these were not detected in our samples (Supplementary Materials 1). Probe sets that were systematically absent (based on the MAS 5.0 detection call) across all the arrays were also removed leaving 37,231 out of the original 45,101 probe sets. General linear models were run on the 134 arrays using drug, stress and strain as fixed effects and sex and dose (acute and chronic) included as covariate in the model. Analysis of Covariance (ANCOVA) did not reveal a significant effect of these two covariates. Any variance explained by these two factors was removed using linear regression and residuals were carried forward for analysis. For comparison purposes, we conducted a traditional linear association analysis using expression as predictor and drug class as outcome. Only one probe‐set associated with drug treatment survived FDR correction for multiple testing. This result was used to provide a comparison baseline to the methods used in this study.

## MACHINE LEARNING APPROACH

### Classification

In statistical learning, a classification task is one wherein we have a dataset $S={\left\{\left(x_{i},y_{i}\right)\right\}}_{i=1}^{N}$, where N is the number of samples, $x_{i}\in X^{M}$ contains the characteristics of the sample i, and $y_{i}\in Y$ is the class to which the sample belongs. In this study, we explored both a multi‐class and a simpler dichotomous method using binary classifiers and the one‐versus‐all strategy, which consists in training n different classifiers, where n is the number of classes. For each class, a classifier is trained with the samples belonging to that class as positive and the rest as negative. Once the n classifiers are trained, a sample is assigned to the class which gets the largest confidence. For analysis we used the Python package Scikit‐learn, which fits the provided data relying on Regularized Logistic Regression, using Stochastic Gradient Descent (SGD) [Pedregosa et al., 2011].

### Logistic Regression

Logistic Regression is a probabilistic classifier model that fits a vector of coefficients so that the computation of a logistic function gives the probability of a sample belonging to a certain class. For logistic regression the model can be defined as:

$$L\left(y,f\left(x\right)\right)=y\,\log p_{1}(x;w)+(1-y)\log\left(1-p_{1}\left(x;w\right)\right).$$

### Support Vector Machines (SVMs)

SVMs with soft margins and Radial Basis Functions (RBF) are commonly used for classification purposes. SVMs classify data by finding an optimal separating hyper‐plane between data points of different classes [Boser et al., 1992]. SVMs are effectively binary classifiers which in this study have been extended to address a multi‐class problem using the one‐versus‐all method. This method was chosen for interpretability of results as each class is represented by one classifier only; knowledge on membership to a class can simply be obtained by inspecting the classifier.

### l2, l1, and Elastic Net Regularization

A traditional logistic regression model works as a loss function to be minimized when fitting the model. However, it is possible to introduce additional penalty terms to obtain penalized complex models. The ℓ_1_—Norm Regularization (ridge) model used, computes the squared magnitude of the coefficients vector in the Euclidean space.

$$\Omega \left(w\right)=∥w{∥}_{2}^{2}=\overset{M}{\underset{i=1}{\Sigma }}w_{i}^{2}$$

and reduces the magnitude of the coefficients [Hoerl and Kennard, 1970]. When closely correlated features exist, they are assigned similar coefficients, so that the correlated values are averaged. The ℓ_1_—Norm Regularization (lasso)

$$\Omega \left(w\right)=∥w{∥}_{1}\overset{M}{\underset{i=1}{\Sigma }}\left|w_{i}\right|$$

enforce coefficients of irrelevant features to be exactly 0. Therefore, it implicitly discards irrelevant or redundant features. ℓ_1_−Norm Regularization has an important limitation: in the case MN, at most N features are selected. From a group of highly correlated features,as in probe sets, it tends to select only one, dropping features instead of averaging them. The Elastic Net Regularization [Zou and Hastie, 2005] combines both ℓ_1_ and ℓ_2_ norms giving the penalization term

$$\Omega (w)=\alpha ∥w{∥}_{1}+\frac{1}{2}\left(1-\alpha \right)∥w{∥}_{2}^{2},$$

where α regulates the balance between the two norms. The two penalties have different effects. On the one hand, ℓ_1_ norm encourages irrelevant feature weights to be zero acting as feature selection. On the other hand, ℓ_2_ norm penalty produces weight vectors with smaller values. Balance between both penalties is achieved through methods including cross validation.

This type of regularization seems to be a good choice for the kind of data used in this project from a theoretical perspective. The reasoning driving this approach is that genes belonging to biological pathways interact with signals and with each other (epistasis), to carry out their tasks; this scenario is a good match to the advantages of the Elastic Net regularization method [Zou and Hastie, 2005].

### Feature Selection and Filters

Feature Selection is the process whereby only a subset of the original features are kept, reducing the complexity of the data space to explore. Four different filters were used: InfoGain, mRMR, ANOVA, and Chi Squared. For each of these filters the following procedure was undertaken: 10% of the data are held out for testing; the remaining 90% is discretized (except for ANOVA) and used to run the filter and obtain a ranking of the features. Subsequently, different classifiers were trained with varying number of the best scoring features and then tested on the held out data. For training data sets, we performed a 10‐fold cross validation grid search to find the best set of parameters and then fitting the model. The computation of Information Theory measures requires discrete data which we performed in two ways: Naive discretization achieved by splitting the data in 10 equal bins from the minimum value to the maximum and by expression‐based discretization, which is based on the concepts of over and under expression of genes. Values were assigned to −1, 1, or 0 depending on whether it was under/over expressed or not significantly differentially expressed, respectively. A gene was considered over expressed if it was more than two standard deviations away from the mean expression across samples in a positive direction. Analogously expressions falling in the negative side were deemed under expressed. The expressions lying close to the mean were considered not differentially expressed.

### Recursive Feature Elimination (RFE)

RFE (Algorithm 1) is an iterative method that begins by fitting a model with all of the features. It then removes the features with small contributions and refits the model with the remaining features and recursively continues this process until a minimum of features remains [Guyon et al., 2002]. This strategy was used to select a desired number of features and choose the subset size which minimizes the error. We applied RFE in combination with both the SVM and regularised logistic regression.

**Algorithm 1:** Implementation of the RFE algorithm. The Ef (w) is representing the loss function to optimize for the classifier

**Input:** k Number of features to select

**Input:** m Step

**Input:** f Classifier

**Input:**

S = {All features}

**while** S ≥ k **do**

**W**←argmin_w_ϵ_f_ (**w**)

****S←S\{Features with smallest coefficient sin **W**}

****S_S_←S

**end while**

**return** S_k _⊂ S_k+m _⊂ S_k+2m _⊂… ⊂ S_M_

### Evaluation Metrics and Features Comparison

Classification accuracy, expressed as percentage of samples correctly classified, on testing dataset or following Stratified Cross Validation were used as assessment metrics [Braga‐Neto and Dougherty, 2004]. Jaccard distance, which indexes the degree of similarity between two sets of features, was used to compare the selected subsets of features to ascertain the degree to which they were consistent across the different methods used.

$$d_{J}(A,B)=\frac{\left|A\cup B\right|-\left|A\cap B\right|}{\left|A\cup B\right|}.$$

The Jaccard distance can take values in the interval [0,1], being 1 for disjoint sets and 0 for equivalent sets.

### Gene Networks and Pathway Analysis

MetaCore™ (https://portal.genego.com/) was used to explore the molecular association between genes lists obtained from the two binary comparison (escitalopram vs. control and nortriptyline vs. control) and known pathways maps and functional networks. MetaCore™ scores and prioritizes networks and pathways based on the relevance of the gene sets uploaded. The software evaluates the magnitude of the intersection between the uploaded genes and the set of genes corresponding to a network module using different statistical metrics including *P* values and G‐scores. *P* values are calculated based on hypergeometric distributions and used to establish whether saturation with the genes of interest is higher than random. When exploring signaling cascades, this allows one to evaluate if a network contains any fragments of well understood (canonical) signaling pathways. The G‐score is another metric used by MetaCore™ that effectively modifies the Z‐score based on the number of the linear canonical pathway fragments contained within the network. A network highly saturated with reference genes and containing several canonical pathways will achieve a higher G‐score. In this study we have explored the top ranking networks by *P*‐value and by G‐score and reported the highest associated functional pathway. These pathways were also compared to understand potential dissimilarities between the two drug comparisons.

## RESULTS

### Multi‐Class Comparisons and Methods Selection

The first analysis empirically evaluated the performance of several statistical learning methods with different levels of feature selection in a 3‐way, multi‐class design with two drug groups and a control group. Given the three possible decision class at outcome (nortriptyline, escitalopram, and saline), chance levels were considered at 33.3%. The results show that support vector machines with InfoGain using naive discretization was able to classify animals by treatment group above chance levels and outperformed both ℓ_1_ and ℓ_2_ fnorm regularization methods and Elastic Net, which clearly yielded poor results (Table I). Indeed, the classification of animals by drug treatment condition seemed sensitive to the statistical methodology used.

**Table I:** Summary of Results for the Multi‐Class Classification (Nortriptyline, Escitalopram, and Saline) Analysis

	EN	SVM	L1‐SVM
Probesets	IG D2	IG D1	ANOVA
37,231	0.41 ± 0.12	0.56 ± 0.08	0.44 ± 0.12
30,000	0.40 ± 0.11	0.55 ± 0.09	0.44 ± 0.14
10,000	0.43 ± 0.10	0.54 ± 0.06	0.40 ± 0.14
1,000	0.42 ± 0.12	0.51 ± 0.10	0.40 ± 0.12
100	0.30 ± 0.14	0.48 ± 0.13	0.42 ± 0.12
10	0.29 ± 0.11	0.34 ± 0.13	0.36 ± 0.14

The table reports the three top performing methods and combination of feature selection of discretization. Values represent accuracy scores with corresponding errors terms. Given that this is a 3‐way multiclass problem, chance accuracy is considered at 33%. The first column reports the number of probesets used for classification following feature reduction. The second column shows the results from the Elastic net regularization method, with InfoGain and D2 discretization. The third column reports the results using Support Vector Machines with InfoGain and D1 discritization. Finally the last column shows the results from the combination of ℓ_1_—norm regularization method with SVM with ANOVA for feature reduction. The best classification performance was achieved with and SVM with infogain and D1 (naive‐discretization).

Across all methods used, the results get progressively weaker as we reduce the number of predictive features. This suggests that the molecular perturbations caused by antidepressant drugs are sparsely distributed across a very broad genomic region, pointing at the highly polygenic nature of AD response. It is likely that AD drugs affect in excess of 5,000 genes by a very small amount, which may explain the generally weak results reported using traditional linear association methods. The strongest classification considering error terms was achieved with the inclusion of 10,000 probe‐sets. Below 1,000 features none of the methods was able to classify animals into treatment group above chance levels in a 3‐way multi‐class design.

### Binary Classifications—Drug Versus Control

Differences between treated and control animals were also explored as part of a simpler, two‐class solution for each of the two drugs separately. The first binary analysis used mRNA expression levels to classify animals into nortriptyline or control groups while the second analysis classified animals into escitalopram or control groups. A summary of results for the nortriptyline versus control group is presented in Table II.

**Table II:** Summary of Binary Classification: Nortriptyline (NRI) Versus Control (Saline)

	EN			SVM‐radial basis			SVM‐linear kernel		
Features	ANOVA	IG 10	IG exp	ANOVA	IG 10	IG exp	ANOVA	IG 10	IG exp
37,231	0.54 ± 0.04	0.51 ± 0.06	0.57 ± 0.08	0.71 ± 0.11	0.76 ± 0.10	0.54 ± 0.10	0.66 ± 0.15	0.72 ± 0.11	0.58 ± 0.15
30,000	0.51 ± 0.06	0.51 ± 0.06	0.57 ± 0.10	0.69 ± 0.16	0.77 ± 0.12	0.54 ± 0.10	0.66 ± 0.15	0.71 ± 0.11	0.58 ± 0.15
10,000	0.49 ± 0.06	0.50 ± 0.06	0.54 ± 0.13	0.69 ± 0.14	0.74 ± 0.12	0.52 ± 0.11	0.69 ± 0.19	0.70 ± 0.09	0.57 ± 0.17
1,000	0.52 ± 0.05	0.52 ± 0.05	0.57 ± 0.12	0.68 ± 0.10	0.67 ± 0.16	0.58 ± 0.14	0.67 ± 0.12	0.69 ± 0.16	0.56 ± 0.18
100	0.51 ± 0.06	0.50 ± 0.06	0.45 ± 0.16	0.73 ± 0.11	0.63 ± 0.12	0.37 ± 0.17	0.69 ± 0.11	0.54 ± 0.11	0.38 ± 0.11
10	0.50 ± 0.06	0.49 ± 0.06	0.48 ± 0.18	0.62 ± 0.16	0.60 ± 0.20	0.34 ± 0.18	0.59 ± 0.17	0.62 ± 0.16	0.46 ± 0.16

EN, elastic net, IG10, InfoGain 10, IG EXP, InfoGain Exponential, SVM, support vector machines with either radial or linear kernel. Values show average accuracy following validation with error terms. The highest classification accuracy (77%) was achieved using an SVM with a radial kernel with InfoGain 10. Elastic net regularization performed poorly. The best accuracy was achieved using in excess of 10,000 probesets. This may suggest that a high number of genes may be implicated in NRI treatment response.

The highest classification accuracy was achieved with an SVM using radial basis function and 30,000 probe sets (77%). The results are consistent with the multi‐class analysis both in terms of number of features likely to be involved and in terms of the statistical methods used. The second binary comparison was conducted on the escitalopram or control groups. The results of this analysis show that it was possible to classify animals above chance level (>67%). However, classification was only possible using feature reduction (the best results obtained using ANOVA) suggesting a less polygenic architecture than what observed with an NRI (Table III). This may be consistent with the more selective targets of SSRI drugs. Similarly to what was observed with the multi‐class analysis, regularization methods including Elastic Net performed sub‐optimally. In both cases, classification above chance levels was achieved but only through careful optimization of the methods. Across all analysis, validation methods were used to ensure models were not over‐fitted to the data.

**Table III:** Summary of Binary Classification: Escitalopram (SSRI) Versus Control (Saline)

	EN			SVM‐radial basis			SVM‐linear kernel		
Features	ANOVA	IG 10	IG exp	ANOVA	IG 10	IG exp	ANOVA	IG 10	IG exp
37,231	0.50 ± 0.00	0.50 ± 0.00	0.52 ± 0.06	0.55 ± 0.12	0.57 ± 0.20	0.59 ± 0.16	0.63 ± 0.13	0.53 ± 0.13	0.60 ± 0.16
30,000	0.50 ± 0.00	0.50 ± 0.00	0.56 ± 0.08	0.57 ± 0.13	0.60 ± 0.18	0.61 ± 0.16	0.61 ± 0.15	0.53 ± 0.13	0.60 ± 0.16
10,000	0.50 ± 0.00	0.50 ± 0.00	0.60 ± 0.12	0.59 ± 0.15	0.57 ± 0.19	0.66 ± 0.18	0.59 ± 0.13	0.57 ± 0.18	0.56 ± 0.11
1,000	0.50 ± 0.00	0.50 ± 0.00	0.52 ± 0.18	0.60 ± 0.16	0.67 ± 0.20	0.58 ± 0.19	0.63 ± 0.15	0.59 ± 0.16	0.50 ± 0.23
100	0.50 ± 0.00	0.49 ± 0.03	0.61 ± 0.13	0.55 ± 0.13	0.62 ± 0.15	0.52 ± 0.15	0.67 ± 0.13	0.52 ± 0.13	0.53 ± 0.17
10	0.50 ± 0.00	0.49 ± 0.03	0.47 ± 0.17	0.57 ± 0.12	0.54 ± 0.15	0.41 ± 0.07	0.61 ± 0.16	0.45 ± 0.18	0.53 ± 0.14

EN, elastic net, IG10, InfoGain 10, IG EXP, InfoGain Exponential, SVM, support vector machines with either radial or linear kernel. Values show average accuracy following validation with error terms. The result from this binary classification are generally weaker than with nortriptyline. The highest classification accuracy (67%) was achieved using a linear kernel and ANOVA as feature reduction method. The elastic net performed poorly across both binary classifications.

### Feature Extraction

The top ranking features based on Jaccard distance were extracted for each of the binary comparisons. A total of 241 probe sets were uncovered from the nortriptyline (NRI) versus control analysis and 204 from the escitalopram (SSRI) versus control comparison. Examining the two sets of probes, only 20 probe sets overlapped across both (<10%) analysis. This finding suggests that the two drugs have distinct molecular mechanisms of action. Indeed, the different gene targets could provide clues on the candidate gene sets that could be used in the search for predictors of differential treatment response in future human studies. In order to gain further biological insight the two gene‐lists were carried forward for pathway and network analysis using MetaCore™.

### Gene Network and Enrichment Analysis

**Figure 1:**
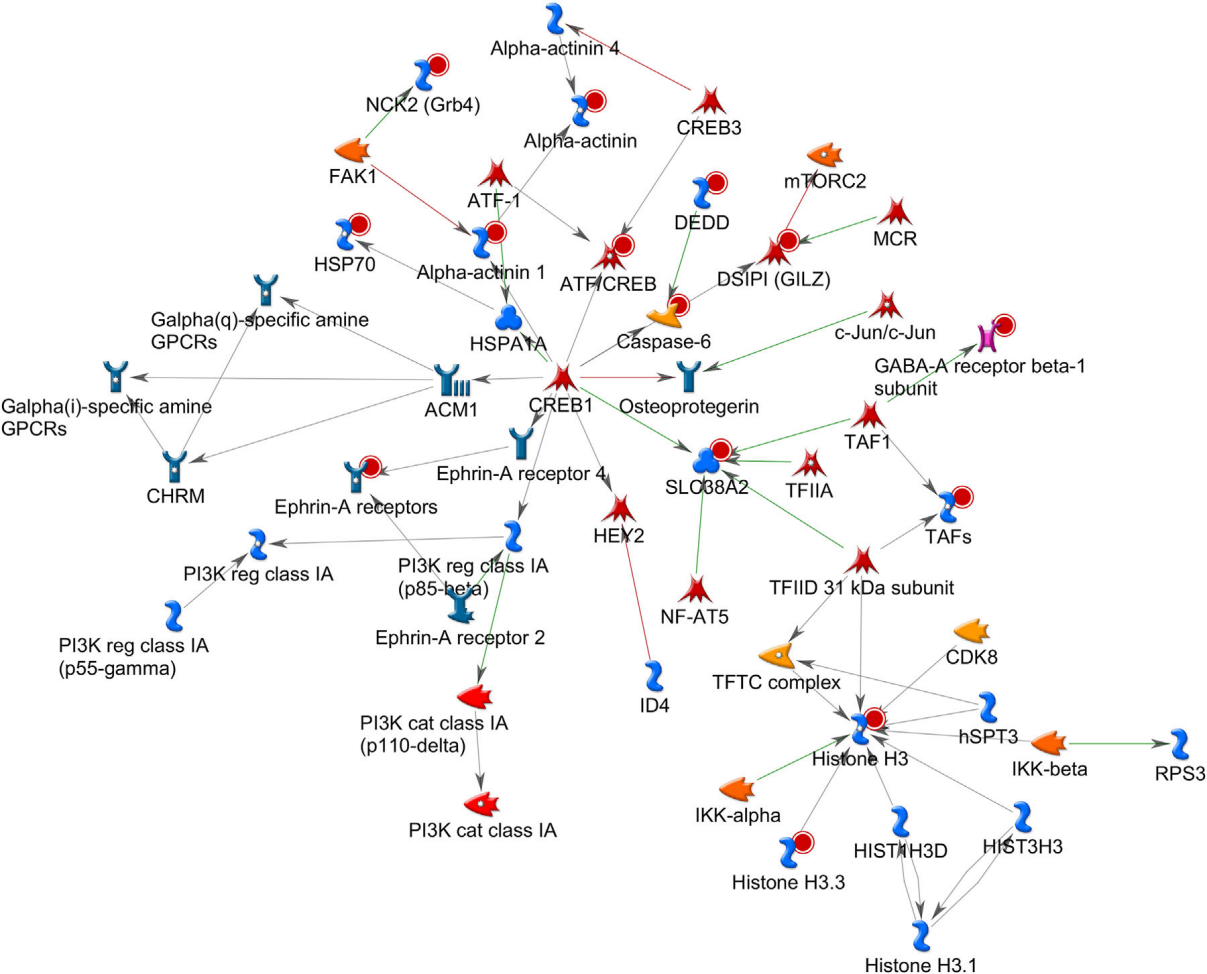
Top ranking gene network by *P*‐value from the gene list uploaded from the NRI versus Control analysis. Uploaded reference molecules are identified with a red circle. The pathway is centered on the *CREB1* gene hub with several seed genes including the *ATF/Creb*, *Casp6*, *Slc38a2*, and *Acta1* genes, interacting directly with it and several others one interaction away. Creb has been systematically found to be activated by AD treatment and is a key mechanism involved in hippocampal neurogenesis and inflammation. A second gene hub based on the uploaded *Hdac3* gene was also uncovered. *Hdac3* inhibitors have been shown to have antidepressant effects in animal models. [Color figure can be viewed at wileyonlinelibrary.com].

The top ranking probe sets extracted from the nortriptyline and control classification analysis were uploaded to MetaCore™ for pathway and enrichment analysis. A total of 241 probe sets from the NRI versus control comparison were matched to 206 genes in MetaCore™ database (Supplementary Materials 2). First, networks were created using uploaded reference molecules as seeds and interactions with molecules in MetaCore™ database as edges. The top scoring networks by *P*‐value and G‐Score are reported. The first pathway, with a *P*‐value of 4.58 × 10^−28^ and G‐Score of 49.06, containing 14 reference molecules was a network centered on the *CREB1* gene complex (Fig. 1). Several of the uploaded genes show a direct interaction with *Creb1*, including *ATF Creb*, *ATF‐1*, *SLC38A2*, Caspase‐6, and several others are one interaction away. The association between *CREB1* and its interaction with *BDNF* and Met variants is well documented. Phosphorylation of *CREB1* results in the synthesis of different proteins that play an important role in neuronal cell functioning. In animal models, increase of *CREB1* has been associated with antidepressant‐ like effects [Chen et al., 2001]. *CREB* mediates the transcription of genes containing a cAMP‐responsive element and is induced by different factors including neurotrophic and inflammatory signals. Inflammatory pathways have been implicated in aetiology in the pathomechanisms of antidepressant efficacy [Chen et al., 2001]. A second gene‐hub centered on the Histone H3 gene, which belonged to the uploaded gene list, is also present in the same network. In mouse, chronic social defeat stress models have been associated with increase in H3 acetylation and decreased levels of histone deacetylase 2 (*HDAC2*) suggesting that antidepressants may also act through *HDAC* inhibition [Covington et al., 2009].

**Figure 2:**
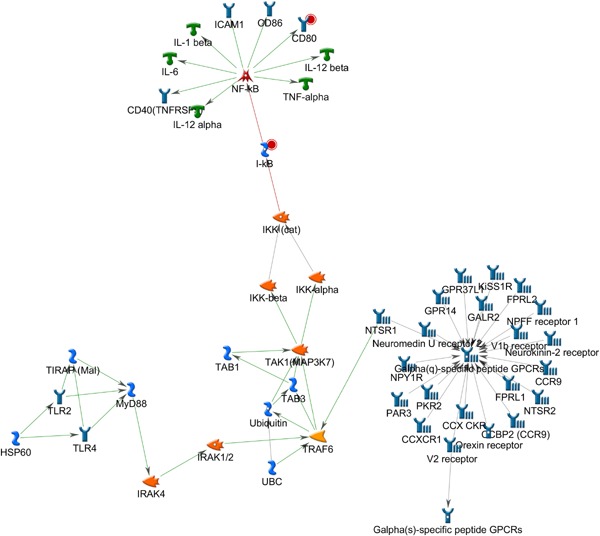
Top ranking gene network by gScore from the gene list uploaded from the NRI versus control analysis. The pathway is highly enriched with fragments of canonical pathways and shows a gene‐network centered on the NF‐κB gene complex. NF‐κB is modulated directly by CREB suggesting an association with mechanisms involved in modulating inflammatory response. [Color figure can be viewed at wileyonlinelibrary.com].

A network highly enriched with canonical fragments with a gScore of 546.777, *P* < 3 × 10^−03^ was also uncovered (Fig. 2). The network is centered on the NF‐κB gene hub with two reference molecules, CD80 and IκB interacting directly with it. Activation of CD80 is associated with T‐cell proliferation and cytokine production, while IκB is an inhibitor of the NF‐κB. Stimuli,such as those induced by cytokins IL‐1 can dissociate IκB from the NF‐κB complex making it available to translocate to the nucleus. The NF‐κB hub and reference molecules play a critical role in limiting cytokine induced cell death. This pathway also suggests an association with inflammation.

**Figure 3:**
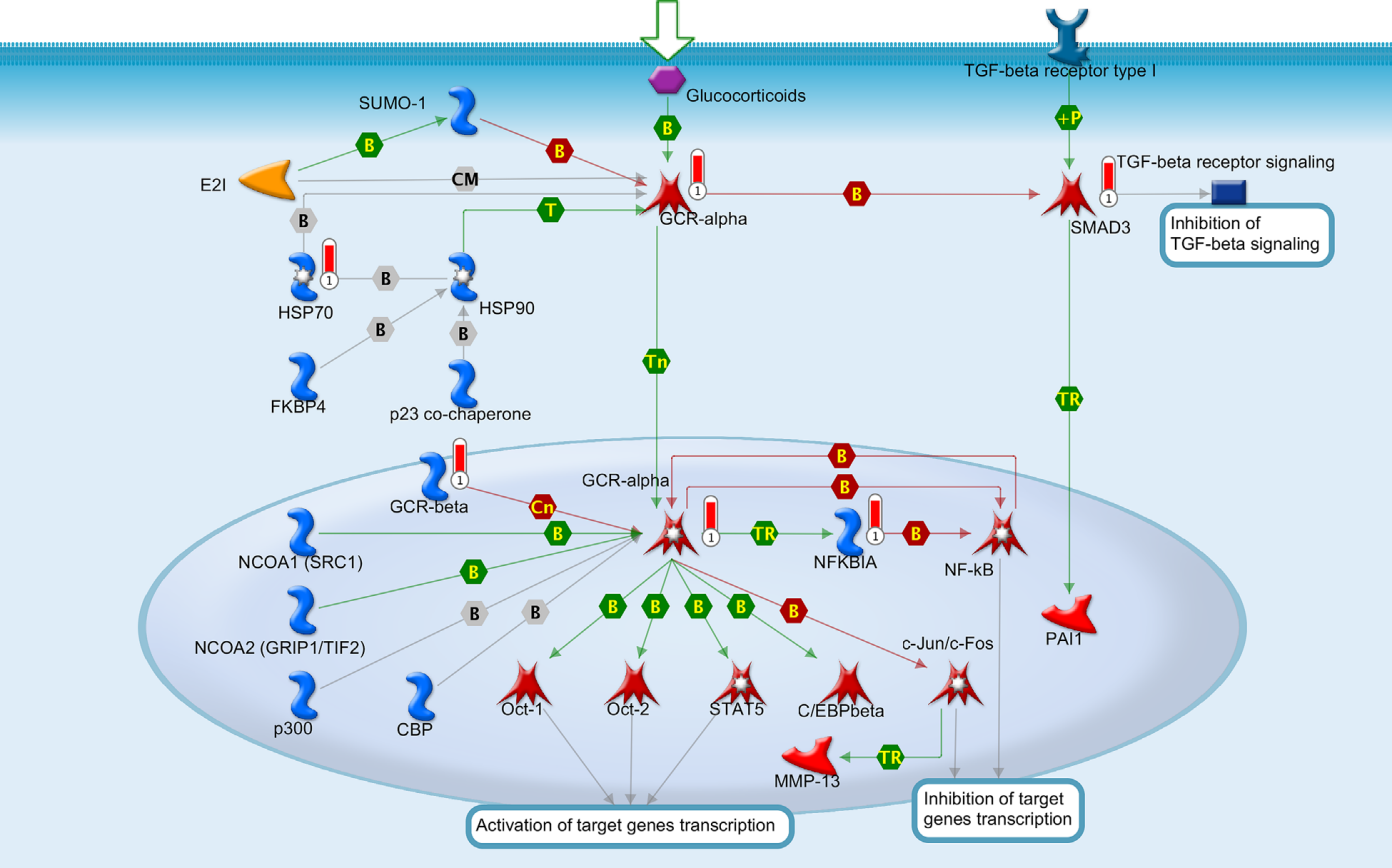
Association between the gene list and known processes pathways reveals a role for Glucocorticoids. These are commonly used for the suppression of inflammation in chronic inflammatory diseases. The convergent evidence across the top‐ranking canonical, network and process pathway suggest a role for NRI in the modulation of inflammatory responses. [Color figure can be viewed at wileyonlinelibrary.com].

Lastly gene set enrichment analysis was used to look for the association between the gene list and known process pathways. The top ranking process pathway *P* < 5.9 × 10^−7^, (FDR corrected, Q < 4.15 × 10^−04^) is significantly associated with development of glucocorticoid receptor signaling (Fig. 3). Glucocorticoid family of genes, including GCR‐α are associated with suppression of NF‐κB by increasing expression of IκB which, as discussed previously, is a potent NF‐κB inhibitor. Glucocorticoids are well‐characterized and potent anti‐inflammatory and immunosuppressive agents, inhibiting all known cytokine synthesis involved in pro‐inflammatory processes [Almawi and Melemedjian, 2002].

**Figure 4:**
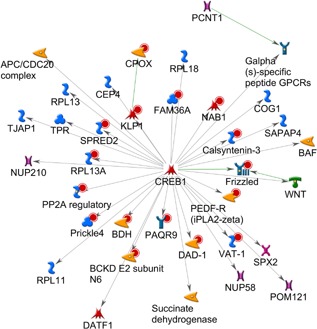
Top ranking gene network by *P*‐value from the gene list uploaded from SSRI versus control group analysis. The pathway is centered on the CREB1 gene hub with all uploaded genes interacting directly with it except one gene that is one interaction away. As with the NRI pathway, there is a strong association with inflammatory processes. Although there is minimal overlap between the genes uncovered across the two analysis both drugs seem to affect the same networks but through different mechanisms. The Frizzled gene, a GWAS hit for schizophrenia, was among the uploaded genes and also shows a direct interaction with *Creb1*. [Color figure can be viewed at wileyonlinelibrary.com].

**Figure 5:**
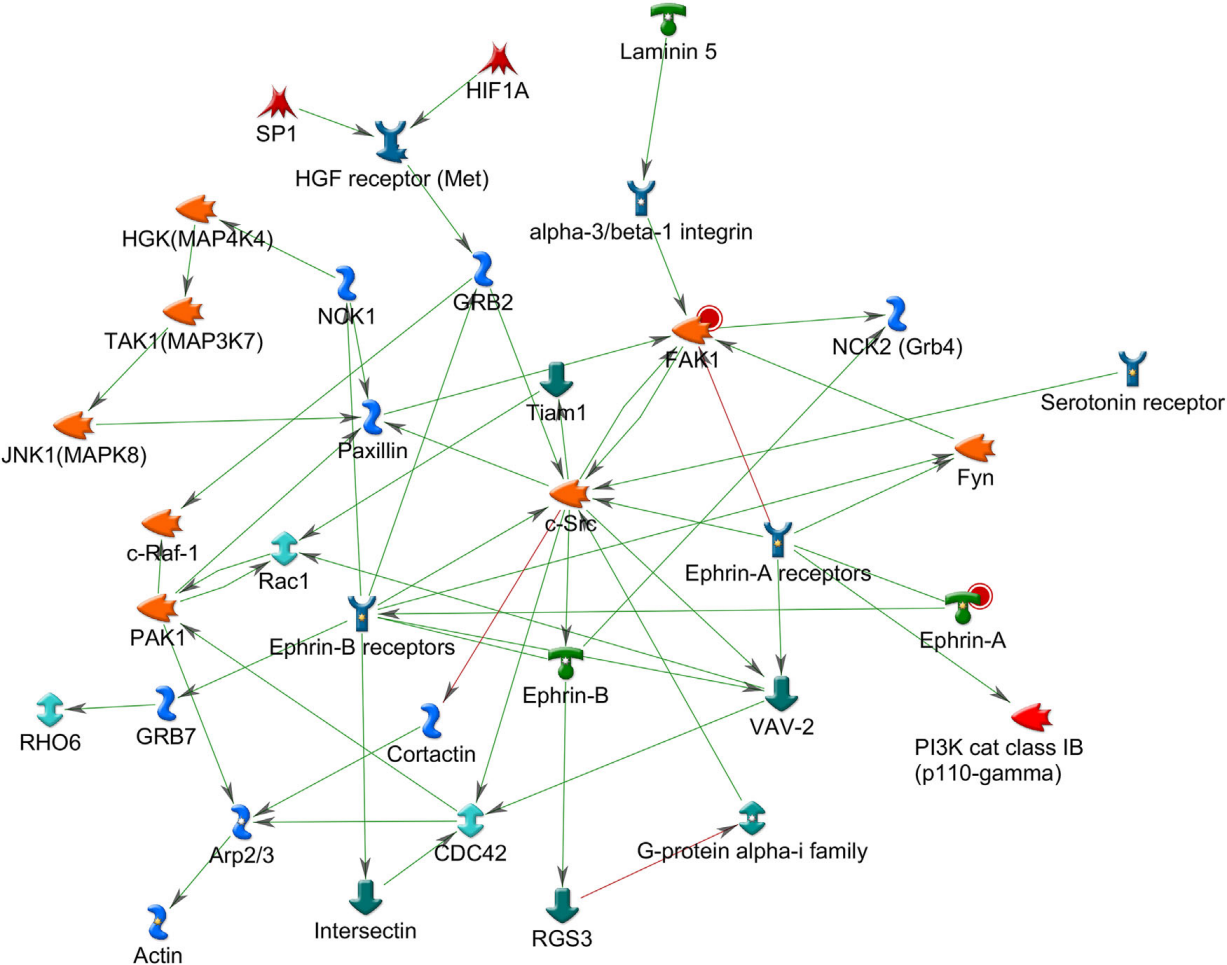
Top ranking gene network by gScore from the gene list uploaded from the SSRI versus control analysis. The pathway is highly enriched with fragments of canonical pathways and shows a gene‐network centered on the c‐Src gene complex. Several studies have found an association between Ephrin and antidepressants. Animal studies have found increased levels of ephrinA in response to stress in rats which is rescued by fluoxetine administration. [Color figure can be viewed at wileyonlinelibrary.com].

The same analysis was repeated for probe sets uncovered from the escitalopram versus saline classification analysis. A total of 204 probe sets were identified and matched to 181 genes in MetaCore™ database. The first two networks uncovered were both strongly associated with *CREB1*, *P* < 5.7 × 10^−22^ and *P* < 5.7 × 10^−22^, with 14 seed molecules interacting directly with the *CREB1* gene hub in both cases (Fig. 4). These include: *VAT‐1*, *DAD‐1*, *PAQR9*, *BCKD*, *BDH*, *Prickle4*, *PP2A*, *RPL13A*, *SPRED2*, *KLP*, *FAM36A*, *NAB1*, *CLSTN3*, *PEDF‐R*, and *FZD3* (Frizzled gene previously associated with different psychopathologies, most notably Schizophrenia). Through different mechanisms of action, networks uncovered for both NRI and SSRI are strongly involved in inflammatory processes. The second network, ranked by G‐Score (G‐Score = 1963.40), is an ephrin receptor signaling pathway, centered on the *c‐Src* gene hub (Fig. 5). Three genes from the ephrin family were uploaded as seed molecules. Previous animal studies have found that ephrin genes, including *EphA4* and *EphrinA3*, were deregulated in animal models of depression and that these can be rescued by fluoxetine administration [Xiao et al., 2006]. Pathway analysis from the Star*D study also reported a potential role for the substrate (*EFNA5*) and receptor (*EPHA5*) genes of ephrin‐A5, in cluster of genes potentially involved in SSRI treatment response [Ising et al., 2009]. Similarly a combined phenotype of treatment outcome in the MARS study was found with a SNP located downstream of EPHB1. Animal studies suggest that the ephrin system is involved in the regulation of neural plasticity in the hippocampus. Indeed, previous studies suggested that antidepressants may work through stimulation of neurogenesis in this brain area [Malki et al., 2015].

**Figure 6:**
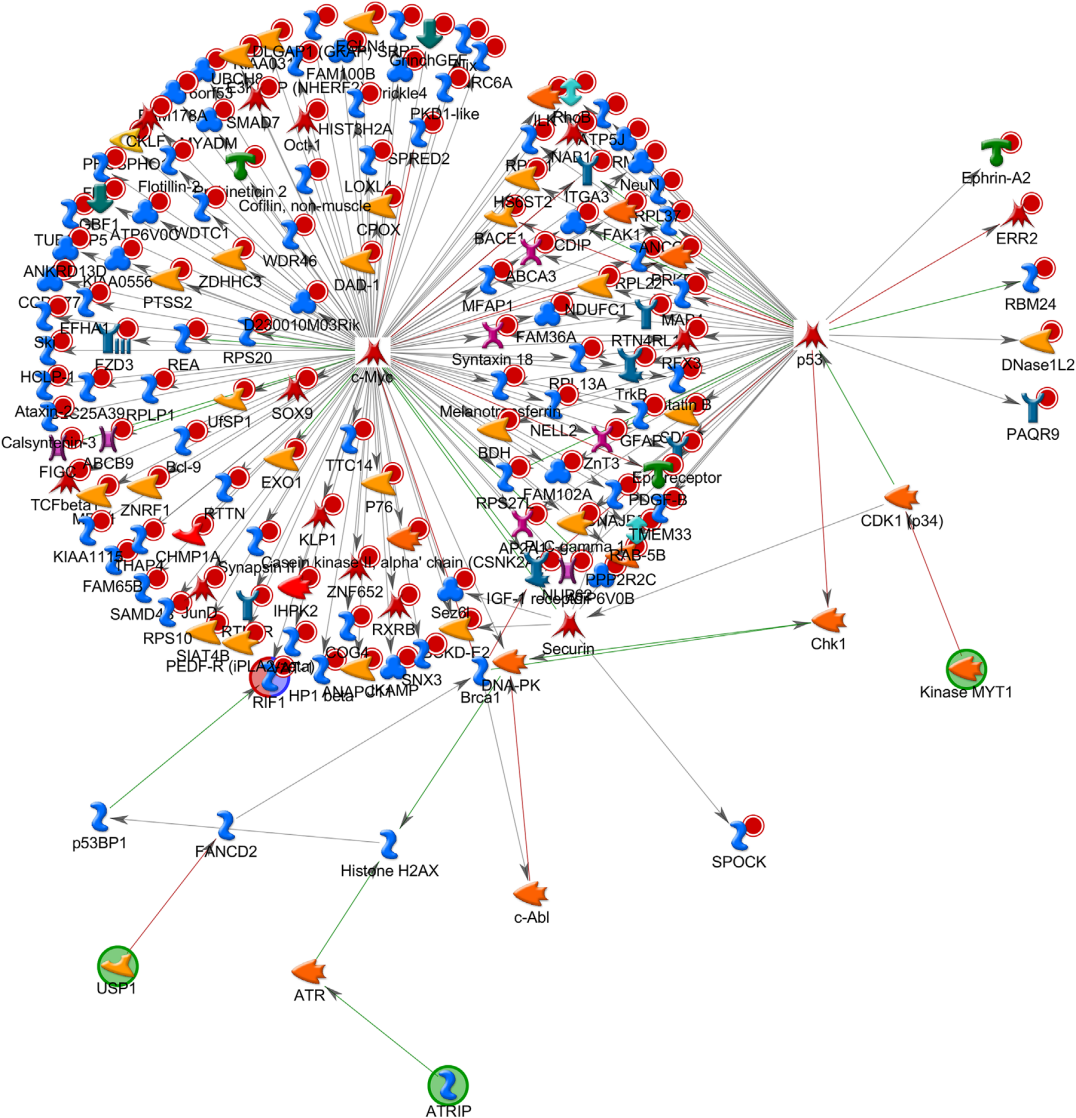
The most significant canonical pathway from the gene list obtained from the esciptalpram versus control analysis. The network includes objects corresponding to the uploaded gene list and other molecules that interact with them and with each other in canonical pathways. The pathway consists of 138 uploaded (seed) genes of which 127 interact directly with the apoptotic signaling by *c‐MYC*. [Color figure can be viewed at wileyonlinelibrary.com].

We further performed a canonical pathway modeling to explore ontology for enrichment (Fig. 6). The top ranking canonical pathway (*P* < 1 × 10^−100^) was associated with cellular organization (*P* < 5.85 × 10^−08^) and biogenesis (*P* < 6.6 × 10^−08^). The pathway consists of 138 uploaded (seed) genes of which 127 interact directly with the apoptotic signaling by c‐MYC. This may further highlight a role for both classes of antidepressants in neuronal cell growth in addition to showing immunological effects.

MetaCore™, allows the comparison of different analyses for similarities and differences. Common processes across the two analysis are related to potential inhibition of TFG‐β signaling (*P* = 1.095 × 10^−2^) and cell regulation and proliferation (*P* = 7.070 × 10^−4^). A network analysis of common objects suggest an association with pathway (*P* = 4.93 × 10^−11^) centered on the C‐Jun gene hub involved the regulation of cell developmental processes and a second equally scored network by *P*‐value associated centered on Ubiquitin (*P* = 4.93 × 10^−11^). Given that both pathways target *CREB1* and that IκB is a targeting mechanism to ubiquitination and subsequent proteasomal degradation, the pathway seems plausible and relevant to the pathways uncovered from both analysis [Taylor et al., 2000].

## DISCUSSION

This study used a statistical learning approach to uncover and compare the genomic signature of response to two antidepressant drugs. The methods of analyses used differ from traditional linear methods that explore the associations between molecular variants and a phenotype using a loci‐by‐loci approach that rank results based on probability thresholds independently of all other variants. The methods presented have two advantages: first, they are able to search for a sparsely distributed signal and capture the predictive effects of many markers simultaneously; second, they allow one to reduce the dimensionality of the data and to identify gene expression changes that are more likely to modulate response to each of the two drugs. The results from our analysis suggest that through distinct molecular mechanisms both drugs appear to modulate the same pathway involving neurotrophic responses and inflammation.

## POLYGENIC ARCHITECTURE OF ANTIDEPRESSANT TREATMENT RESPONSE

Response to drug treatment must be considered the endpoint of pharmacokinetic and pharmacodynamic processes including drug absorption, distribution, interactions, biotransformation, and putative target binding. It is therefore likely that a number of complex biological systems in addition to primary targets are involved in these processes in a time‐dependent manner and that primary drug targets may, in isolation, not be sufficient to account for these processes. Moreover, the time taken by antidepressants to work suggests that additional long‐term modulations of cellular and molecular pathways downstream of target binding are required to induce therapeutic benefits [Malki et al., 2015].

In the current study, mRNA measurements were first used as features to classify animals into drug treatment group or no drug highlighting the differential response to treatment. The results further showed that as the number of features is reduced, classification accuracy decreased to chance levels. The highest classification accuracy was achieved with approximately 10,000 probe sets but the signal remained invariant even using the entire array of probesets. This suggests the absence of a subset of genes of high penetrance and that response to AD may be a trait with a highly polygenic architecture. This is consistent with results from human studies, where no pharmacogenetic associations with clinically meaningful effects have been found [Tansey et al., 2012]. Interestingly, the signal was sensitive to the algorithm used and traditional methods generally preferred for sparse data, including the Elastic net and RFE, did not perform well. Overall, while it was possible to discern a signal above chance levels, the results remain generally weak, particularly for escitalopram (67% classification accuracy). Moreover, the significance of the results must be interpreted in the context of its error, which suggests that classification is just marginally above chance levels.

Binary classification comparing each drug group against control was marginally more accurate for nortriptyline (77%) than for escitalopram (67%). The results are consistent with existing literature showing a stronger association with NRI's rather than with SSRI's. Indeed, the only significant result to date across animal and human studies of AD from the GENDEP study was found with several common polymorphisms within the *PPM1A* gene in response to NRI treatment [Malki et al., 2011]. The results from our study suggest that it is likely that in excess of 1,000 genes may be involved in modulating response to AD, pointing at a complex molecular architecture of AD response.

## TWO AD TREATMENTS SHOW CONVERGING MODES OF ACTION

With only a 10% overlap between probe sets significantly associated with NRI treatment and probe sets significantly associated with SSRI treatment, it would appear the mechanisms of action of two drugs are fairly distinct. Contrary to this, gene set enrichment analysis shows that both the predictors of NRI treatment and SSRI treatment include a network centered on the Cyclic AMP‐responsive element‐binding protein‐1 (*Creb‐1*) molecule. Irrespectively of the distinct gene sets associated with NRI and SSRI treatment, the results point at specific overlaps in which these AD response pathways converge. It is conceivable that differences in these AD response pathways could, in part, be responsible for the differential response to AD treatments. Additionally, the overlap in the AD response pathways could provide insight into important downstream molecules mediating biological processes thought to alleviate symptoms of depression, such as reduced inflammation and neurogenesis as well as being implicated in etiology of the disorder.

*Creb1* is a transcription factor, which upon phosphorylation promotes transcription of genes with a range of functions including neuronal growth, regeneration, synaptic plasticity, and immune function [Mayr and Montminy, 2001; Wen et al., 2010]. Congruent with the findings of this study, *Creb1* has been extensively associated in both the development of MDD and AD response in MDD patients [Dowlatshahi et al., 1998; Zubenko et al., 2003]. Further mechanistic investigations of *Creb1* report it as a converging point of several transduction pathways altered by a range of AD treatments and MDD, such as mitogen‐activated protein kinase and Ca2+/calmodulin dependant protein kinase signal cascades. BDNF (brain derived neurotrophic factor) [Tao et al., 1998], transcriptionally regulated by *Creb1*, is involved in neuronal adaptive responses, neurogenesis and neuronal survival and has been associated with the effects of AD treatment [Ghosh et al., 1994; Tao et al., 1998; Chen et al., 2001; Rossi et al., 2006]. It is therefore likely that BDNF is an important mediator of the association between *Creb1* and AD response. The role of *Creb1* in immune function is also thought to be an important mechanism underlying the well‐established association between inflammation, AD treatment and MDD. *Creb1* promotes anti‐inflammatory processes via a number of mechanisms including the inhibition of NF‐κB activity and induction of IL‐10 expression [Parry and Mackman, 1997; Miller et al., 2009; Saraiva and O'Garra, 2010].

The top ranking pathway by gScore from the NRI versus control comparison is (Fig. 3) centred on the NF‐κB gene hub which complements the Creb1 centred pathway ranked by *P*‐value. Furthermore, pathway analysis highlights genes predicting NRI treatment are primarily under the influence of glucocorticoids (Fig. 4), which have strong anti‐inflammatory effects [Barnes, 1998]. Catecholamines, such as noradrenaline, are released in response to stress which, through the hypothalamic‐pituitary‐adrenal (HPA)‐axis, stimulate the secretion of glucocorticoids. Accordingly, AD treatment has been previously shown to increase the expression of glucocorticoid receptors in the hippocampus [Seckl and Fink, 1992]. Glucocorticoids can inhibit NF‐κB, thus down‐regulating inflammatory responses through the inhibition of its target genes [Mukaida et al., 1994]. AD increase glucocorticoid expression, which inhibit NF‐κB, rendering an anti‐inflammatory effect that could potentially mediate the effects of AD. The glucocorticoid resistance hypothesis for depression suggests that impaired glucocorticoid receptors underlie depression predisposition and could potentially modulate AD response in some individuals. Glucocorticoid receptors also interact with CREB‐binding protein (CBP), which acts as a co‐activator of transcription. CBP also binds other transcription factors that compete for the same binding sites. Studies have shown that depressed patients show higher levels of pro‐inflammatory cytokines, acute phase proteins, chemokines and cellular adhesion molecules [Raison et al., 2006]. Cytokines can activate the HPA axis, causing an elevation of systemic glucocorticoid levels, and at the same time, they can inhibit glucocorticoid receptor function at multiple levels, including the block of its translocation and the induction of isoforms with reduced capacity to bind ligand [Pace and Miller, 2009].

The results suggest an association between the anti‐inflammatory roles of *Creb1* converging mechanisms of action of both classes of antidepressant drugs. NRIs have anti‐inflammatory effect in part due to inhibition of PI3Kσ leading to an increase in *Creb*1 [Mercado et al., 2011].

Predictors of SSRI treatment and predictors of NRI treatment were combined and analysed using gene set enrichment with the aim of highlighting converging biological pathways that underlie both AD response and the aetiology of MDD. Two canonical pathways were returned with equal significance (*P* = 4.93 × 10^−11^), one centered on c‐Jun gene hub, involved in the regulation of cell developmental processes, and the other centered on Ubiquitin, important for protein degradation following oxidation.

## AD TREATMENT SPECIFIC MODES OF ACTION

Although there are significant parallels between the pathways implicated in both AD treatments, there are also interesting differences. These differences could be responsible for the differential response to AD treatments and could be further explored in the search for biomarkers of AD treatment response in future studies. Gene network analysis of SSRI treatment predictors, enriched for canonical pathways, highlighted the importance of the transcription factor c‐Myc. c‐Myc is in part regulated by inflammation and increased oxidative stress.

A pathway highly enriched with canonical fragments that includes several ephrin genes was also associated with escitalopram treatment. Animal models have shown that stress may remodel neuronal dentrites and spines in the hippocampus but that these can be rescued by fluoxetine treatment. In mouse, ephrins are highly expressed in adult hippocampus neurons and play a critical role in neuronal regulation and synapse formation and plasticity [Li et al., 2014]. A human pharmacogenetic association study (MARS) also reported an association between the rs1502174 SNP in the *Ephrin* type‐B receptor gene (*P* = 8.5 × 10^−5^) and responders to SSRI treatment. The study searched for genetic markers for early (2 weeks), late responders and remitters (by week 5). The SNP was associated with all three phenotypes [Ising et al., 2009].

## STRENGTHS AND LIMITATIONS

The study used a bottom‐up data‐driven approach to empirically test different statistical learning methods to capture a shallow and sparse signal at the molecular level driven by AD‐induced perturbation. These methods offer several advantages compared to traditional methods but were both computationally expensive and required careful parameter optimization to avoid over fitting. In this study, we opted to err on the side of caution, sacrificing potentially improved accuracy for robustness of results although this may have increased chances of Type‐II errors.

The study explored transcriptomic profiles in a disease relevant brain region in MDD animal models, which may inform on potential molecular mechanisms involved in the modulation of AD response in humans. However, mice are not miniature human beings and therefore findings require replication in human studies. Indeed there are several aspects of the trait, including environmental interactions, that simply cannot be modeled in mouse. However, this study found support for findings reported from the MARS human pharmacogenetic association study suggesting a role for the ephrin family gene in SSRI treatment response as well as a clear role for inflammatory processes.

Additional methods for features reduction could be explored as part of future studies, which may include SVM‐RFE + mRMR to help overcome some of the limitation of the RFE method which yielded disappointing results. However this highlights the complexity and structure of the signal we are trying to detect. Robust Feature Selection (RFS) and Iterative Feature Perturbation (IFP) could also have been tested [Nie et al., 2010; Canul‐Reich et al., 2012]. A further possibility would be to use grouped LASSO which applies shared penalties to predetermined groups of features. The shape of the data sets used can lead to problems such as data shift induced by train‐test split or class imbalance. Although we have avoided this as much as possible, there exist alternatives to Stratified Cross Validation including Distribution Optimally Balanced SCV (DOB‐SCV) [Moreno‐Torres et al., 2012]. Lastly, methods such as MDLP and experimentation with discretization could be explored further [Rissanen, 1978].

The results may also point at further higher order complex interactions and post‐translational modification that are not captured by microarrays or by the methods used and that should be explored further. Although the assumptions that differential gene expression is associated with drug action is consistent with existing literature, expression changes in brain regions other than hippocampus and in blood have escaped the current investigation [Malki et al., 2016]. Future studies could also explore the integration of ML with convergent methods [Ogden et al., 2004; Le‐Niculescu et al., 2009; Le‐Niculescu et al., 2011].

## CONCLUSIONS

The goal of pharmacogenomics research is to one day be able to predict which dose, of what drug will work for which patient at what time. However, uncovering a biosignature or genetic predictors of sufficient clinical utility to inform prescription of antidepressant drugs for an individual patient remains elusive [Tansey et al., 2012]. A commonly held view was that response to treatment may be a simpler phenotype than the pathology itself and indeed many studies exploring the etiology of MDD have used known targets of therapeutic drugs to inform candidate gene selection. This study adds to a growing body of literature that suggests that response to medication may be a more polygenic and complex trait than previously hypothesized. A deeper understanding of individual differences in AD treatment response should therefore consider the possible interaction between many molecular variants, together with miRNA, DNA methylation, histone modifications and other stochastic factors that may otherwise modulate gene expression. This study further showed that different classes of antidepressant drugs target the same pathway associated with inflammation but through different molecular cascades. Lastly, we found support for the role on the *ephrin* gene in response to SSRI as previously reported in a human pharmacogenetic association study.

## Supporting information

Additional supporting information may be found in the online version of this article.

Supporting Information.

Supporting Information.

Supporting Information.
