# Supplementary material for: Highly polygenic architecture of antidepressant treatment response: Comparative analysis of SSRI and NRI treatment in an animal model of depression
Source: Am J Med Genet B Neuropsychiatr Genet. 2016 Oct 1;174(3):235–50. doi: 10.1002/ajmg.b.32494 (PMC5434854; doi:10.1002/ajmg.b.32494)

Supplementary Material: QC metrics

The first figure shows the distribution of log intensities across the arrays. Probe-set log-intensities are expected to be comparable post-normalisation as visible in our data. The second figure shows a heatmap of correlation coefficients computed for each pair of arrays. Lower coefficients are associated with bigger differences across the arrays. The heatmap is also useful to gain a sense of the homogeneity of the data. The third figure shows a density histogram of log-intensities of all distribution superimposed following normalisation. Overall, the normalisation method worked well across all arrays, returning similar distributions. The following figures are MA plots that show the pair-wise comparison of log-intensity of each array to a reference value, in this case the median array of each group. MA plots are expected to be centred on the Y=0 axis. These are useful to detect intensity-dependent biases. Successful normalisation is assumed to correct for intensity dependent biases. The MA plots for our data look good. The following two graphs show 3'/5' ratio for beta-actin and GAPDH. These appear to be well within normal range. The plots of percentage calls, computed using Affymetrix MAS 5 algorithm was used to flag present probe-sets. Three arrays are on the lower bounds but on closer inspection they are only 1% away from the recommended thresholds; given the large number of arrays this is expected and within tolerance levels. Lastly the Relative Log Expression (RLE) plot shows that all arrays are centred around 0 on a log scale. This is consistent with the underlying assumption as the majority of probes should be invariant.

Boxplot after RMA

Distributions should be comparable between arrays

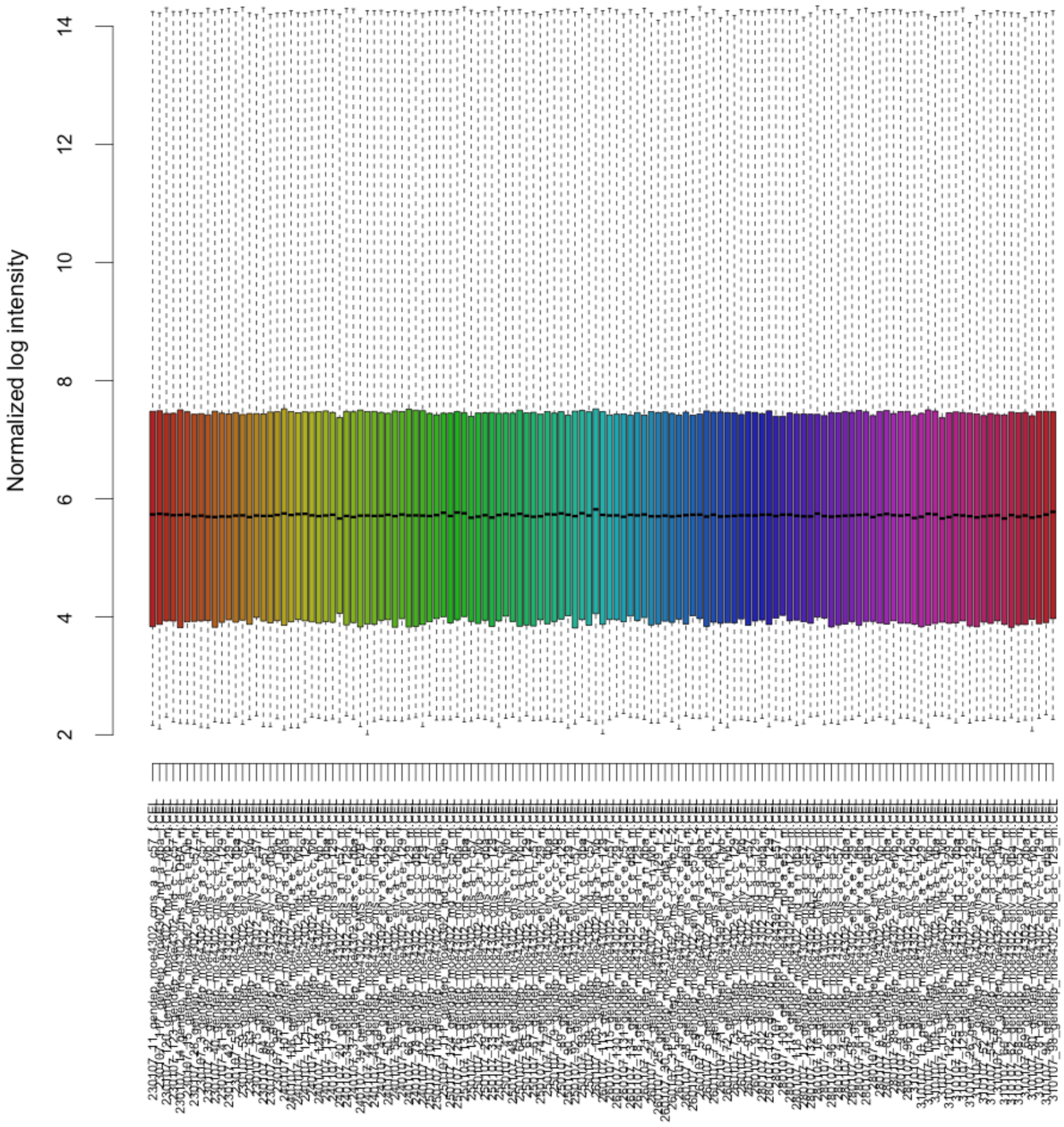

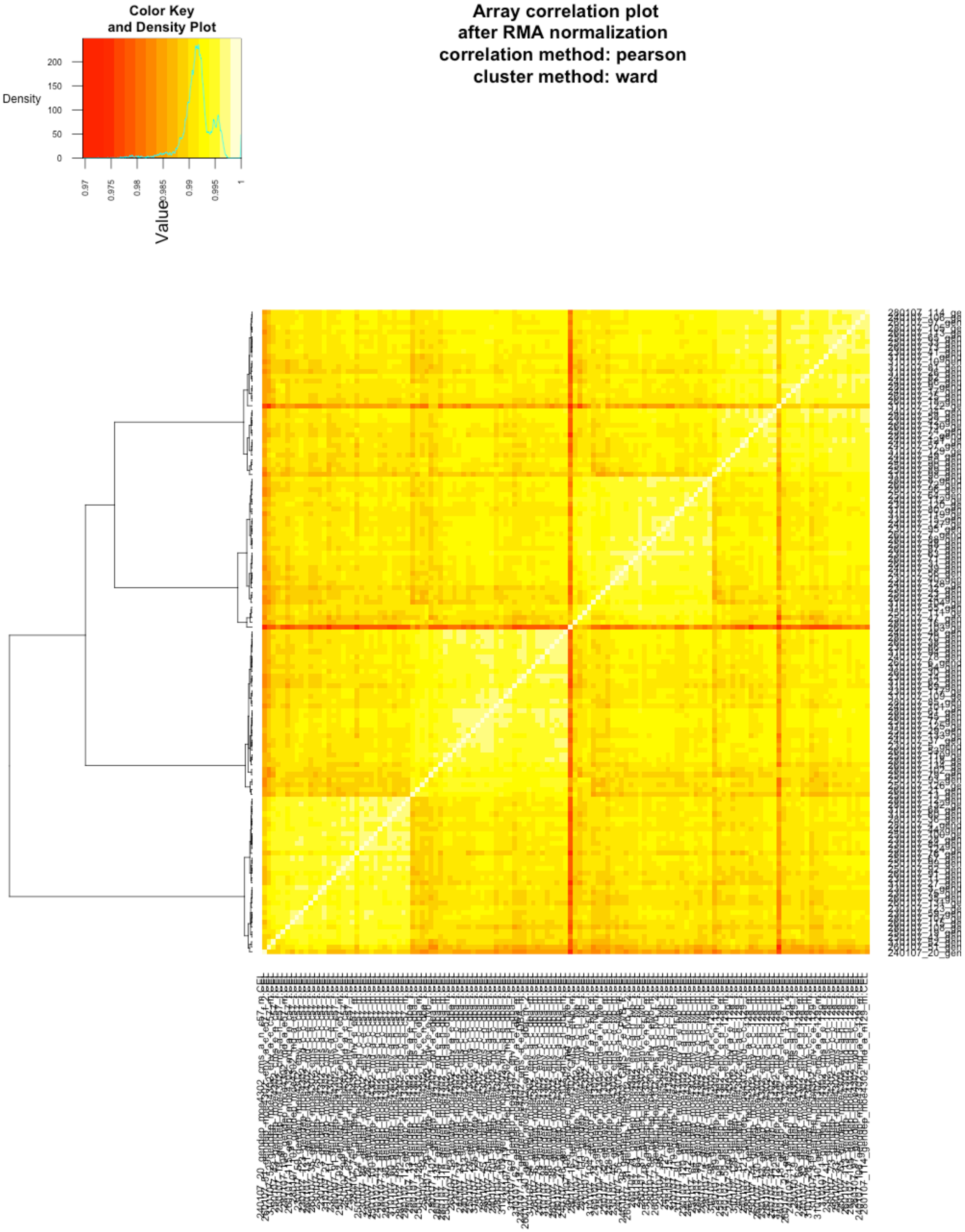
Color Key
and Density Plot
Density
Value
0
50
100
150
200
0.97
0.975
0.98
0.985
0.99
0.995
1
Array correlation plot
after RMA normalization
correlation method: pearson
cluster method: ward

Density histogram after RMA

Curves should be comparable between arrays

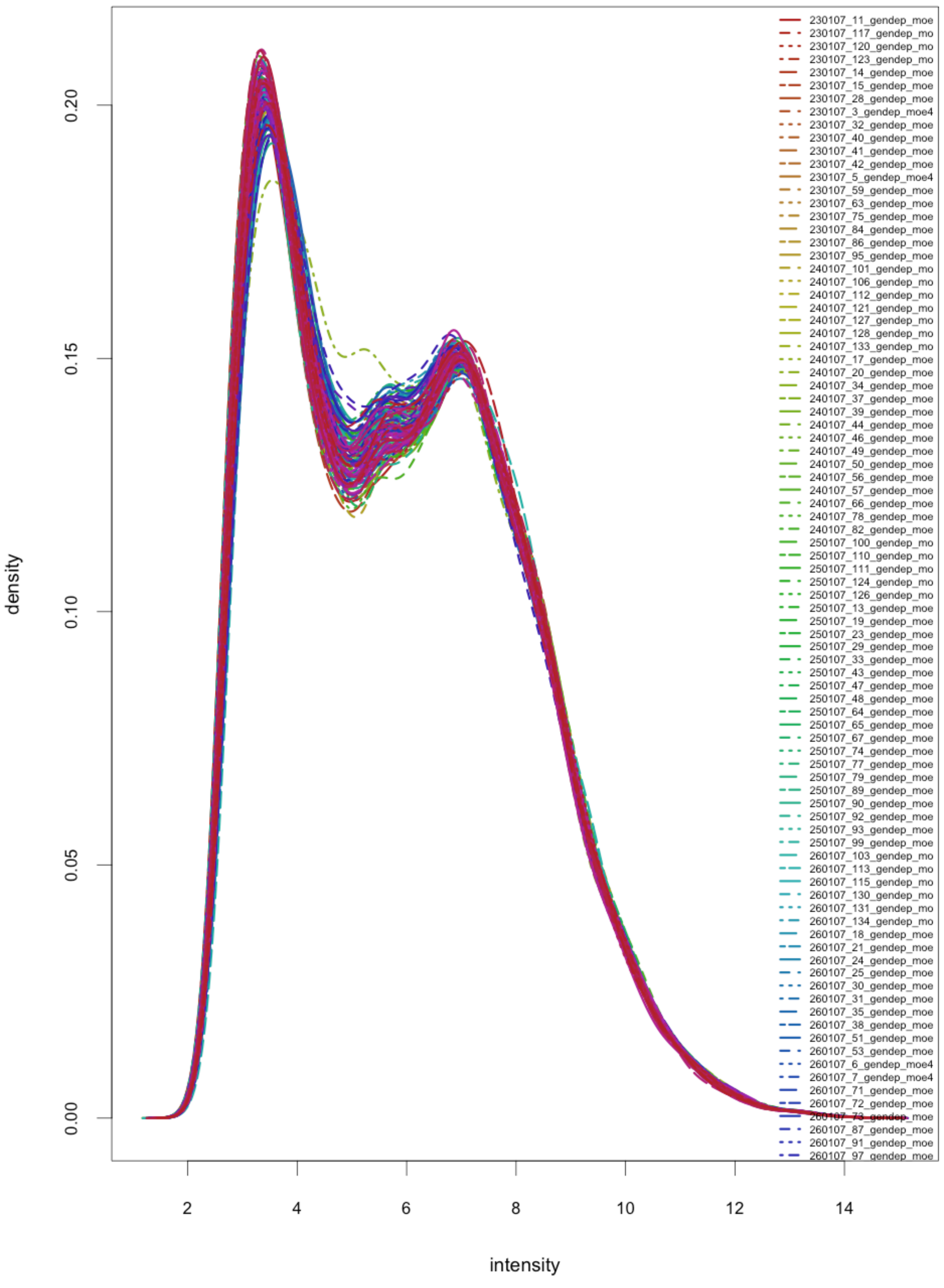

MA plots afterRMAnormalization 1 / 6

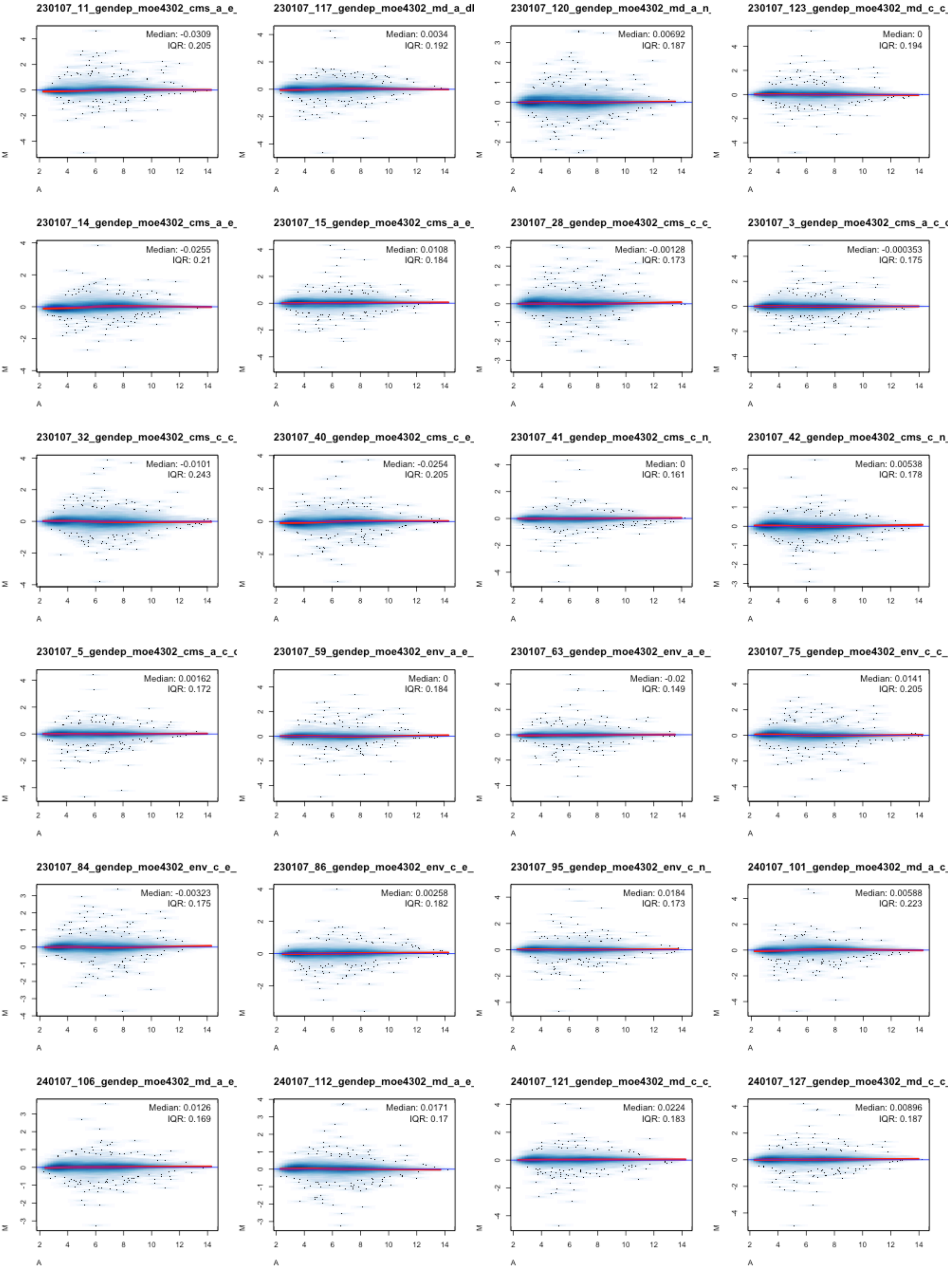

MA plots afterRMAnormalization 2 / 6

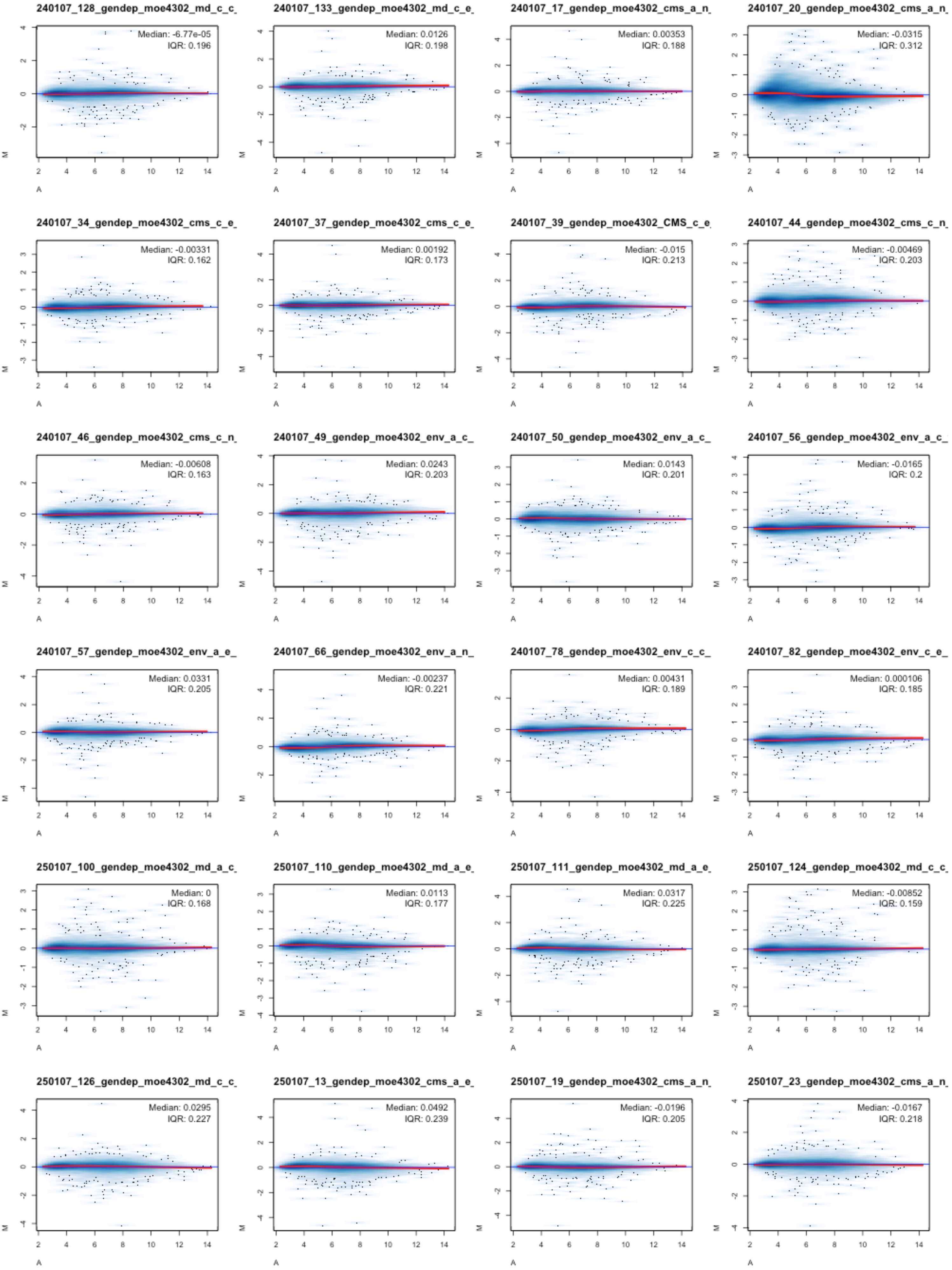

MA plots afterRMAnormalization 3 / 6

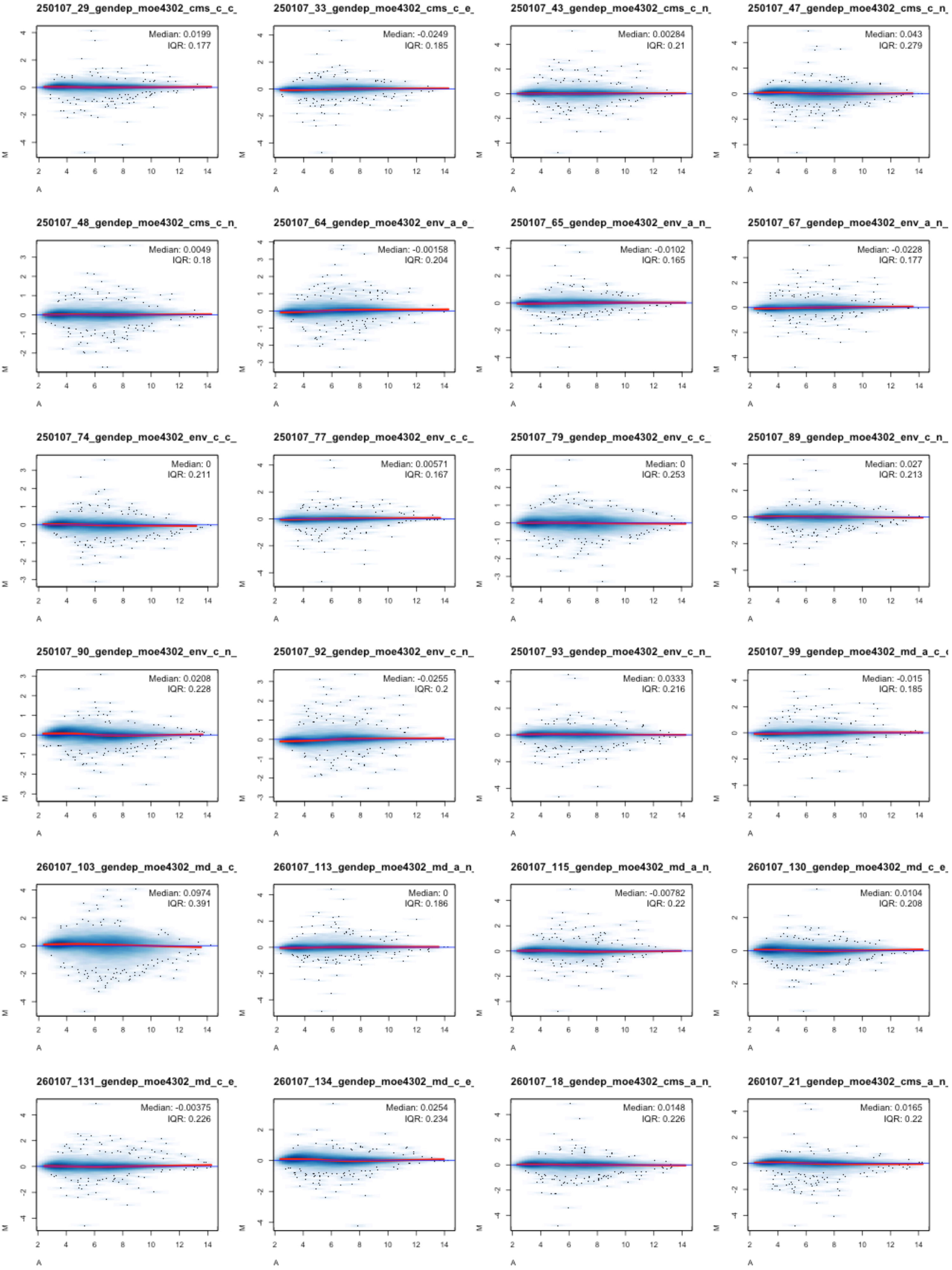

MA plots afterRMAnormalization 4 / 6

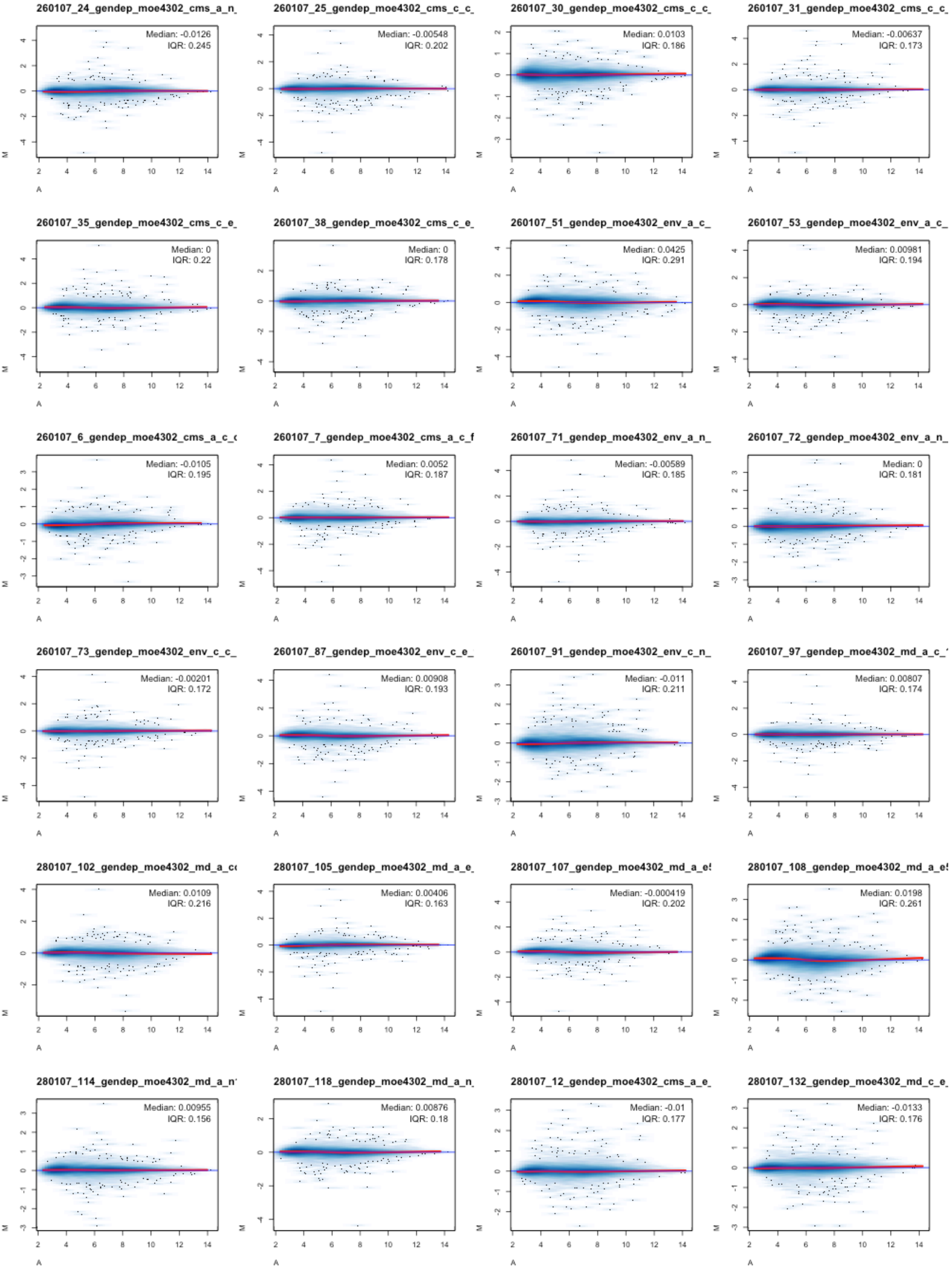

MA plots afterRMAnormalization 5 / 6

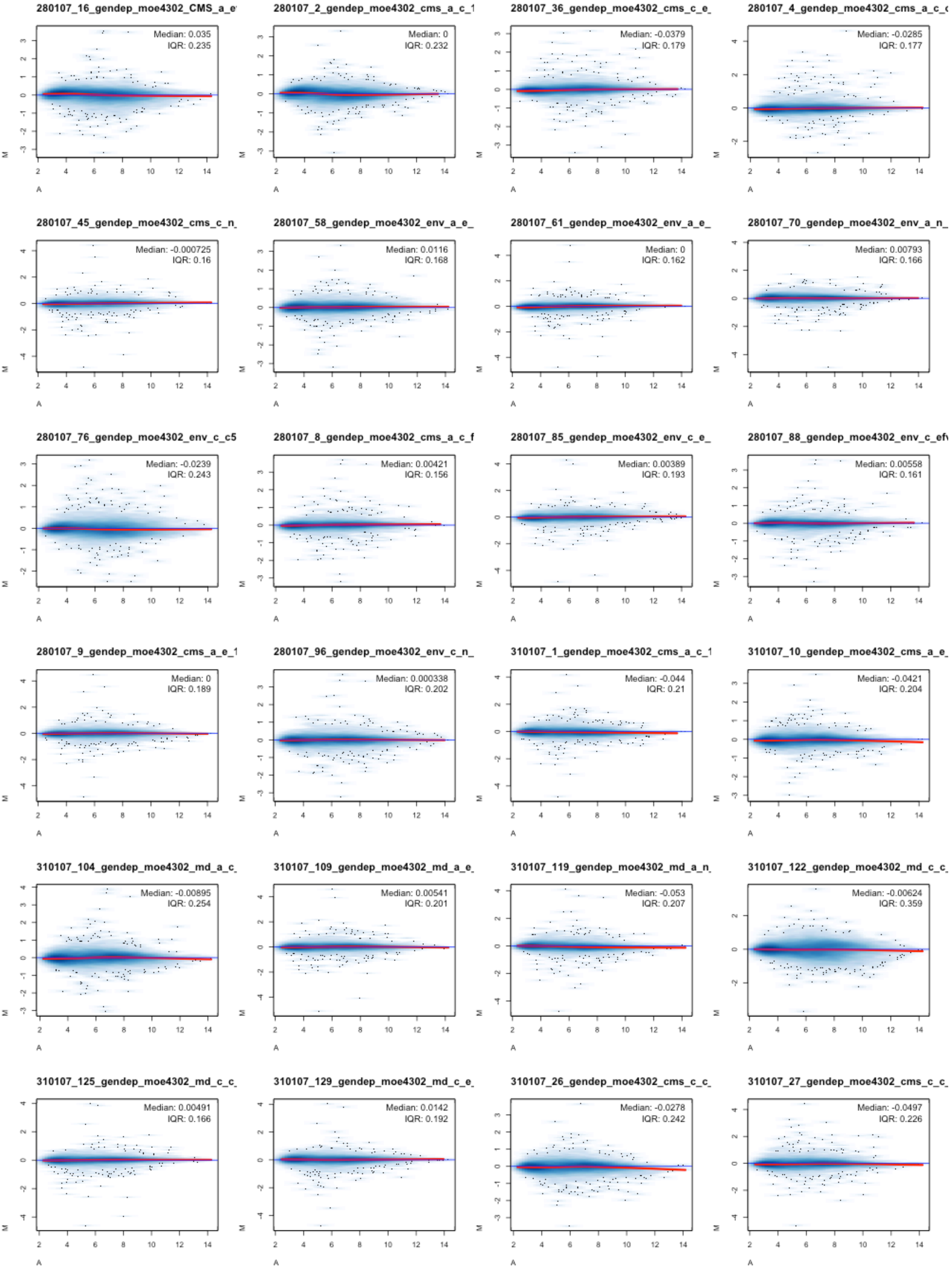

MA plots afterRMAnormalization 6 / 6

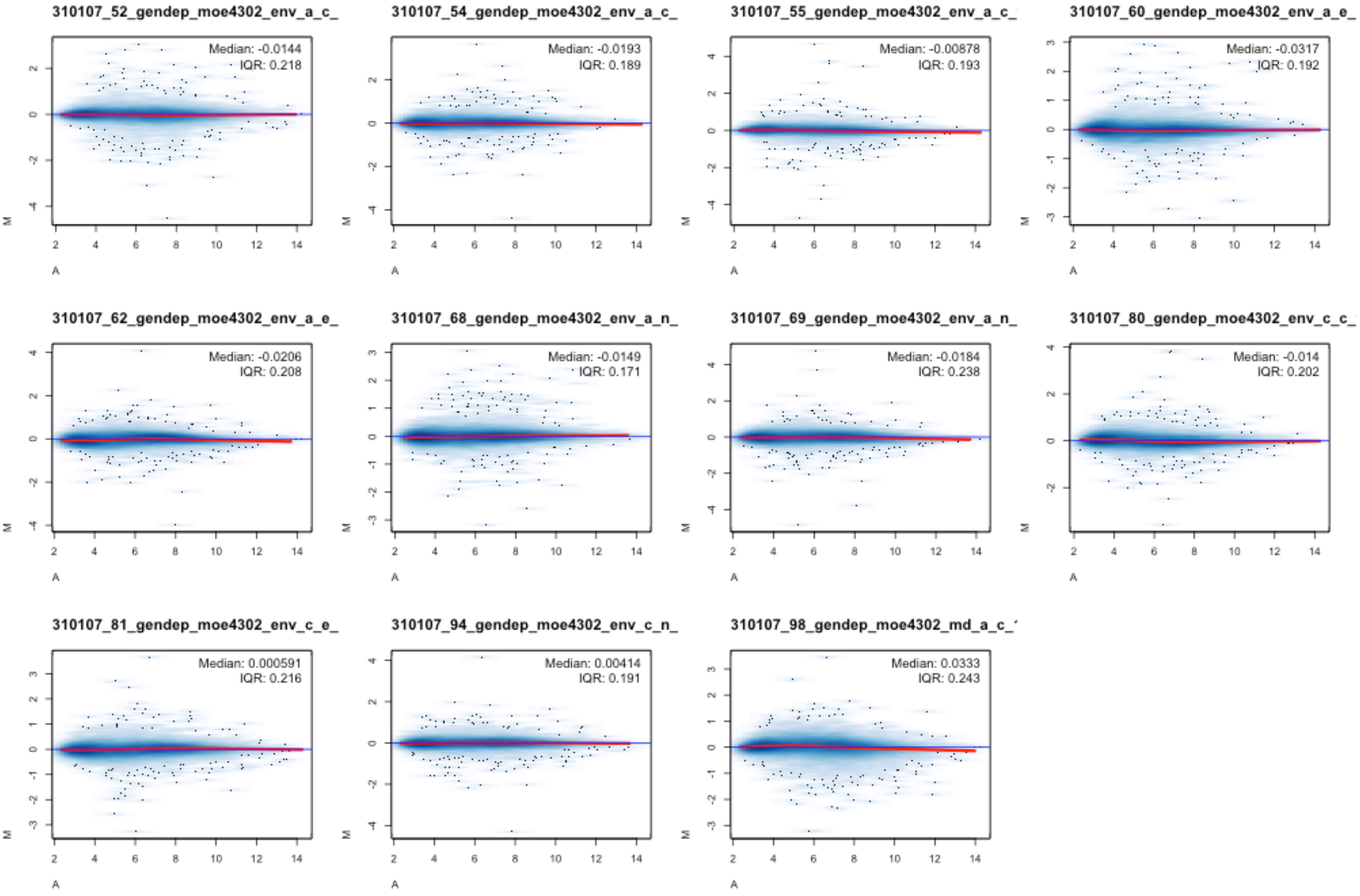

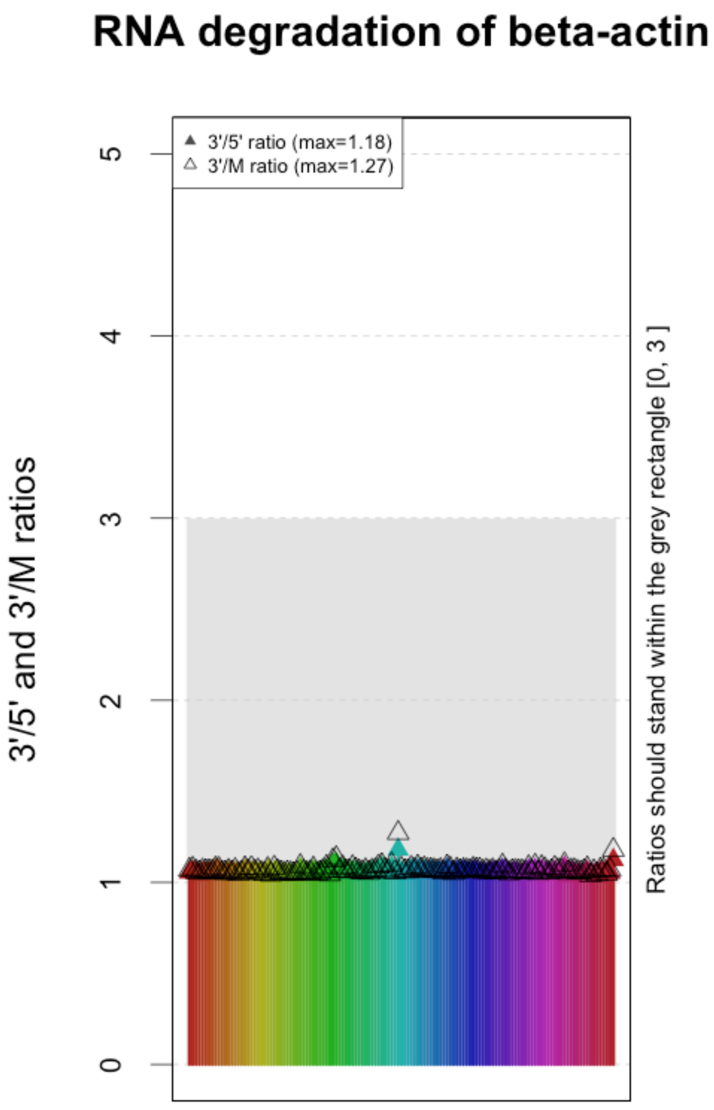

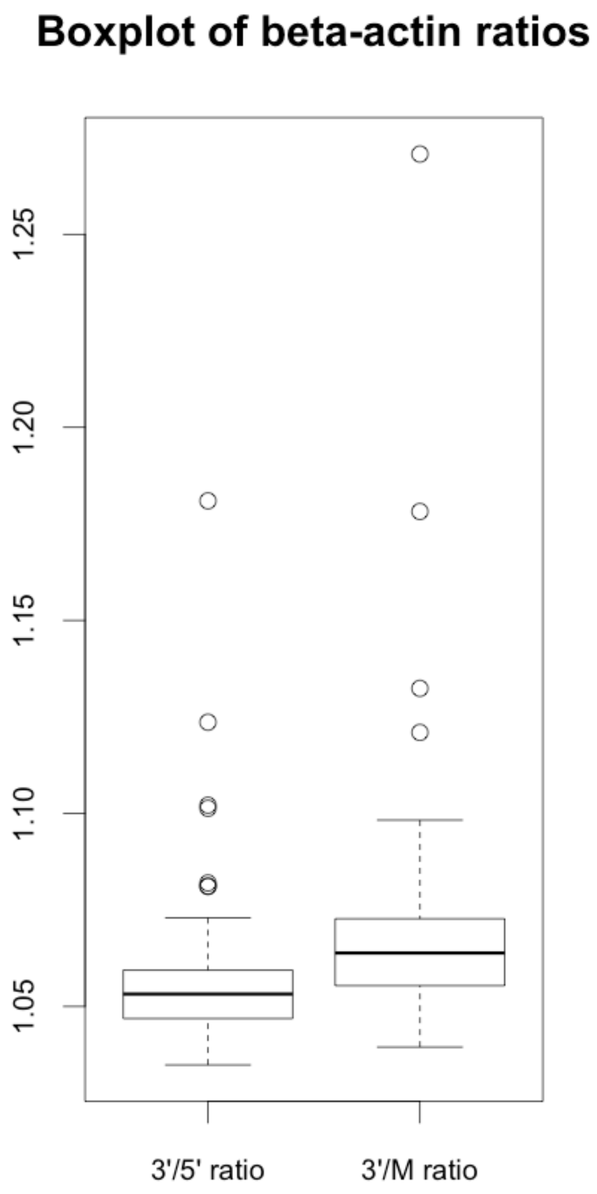

beta-actin QC: OK (all 3'/5' ratios < 3)

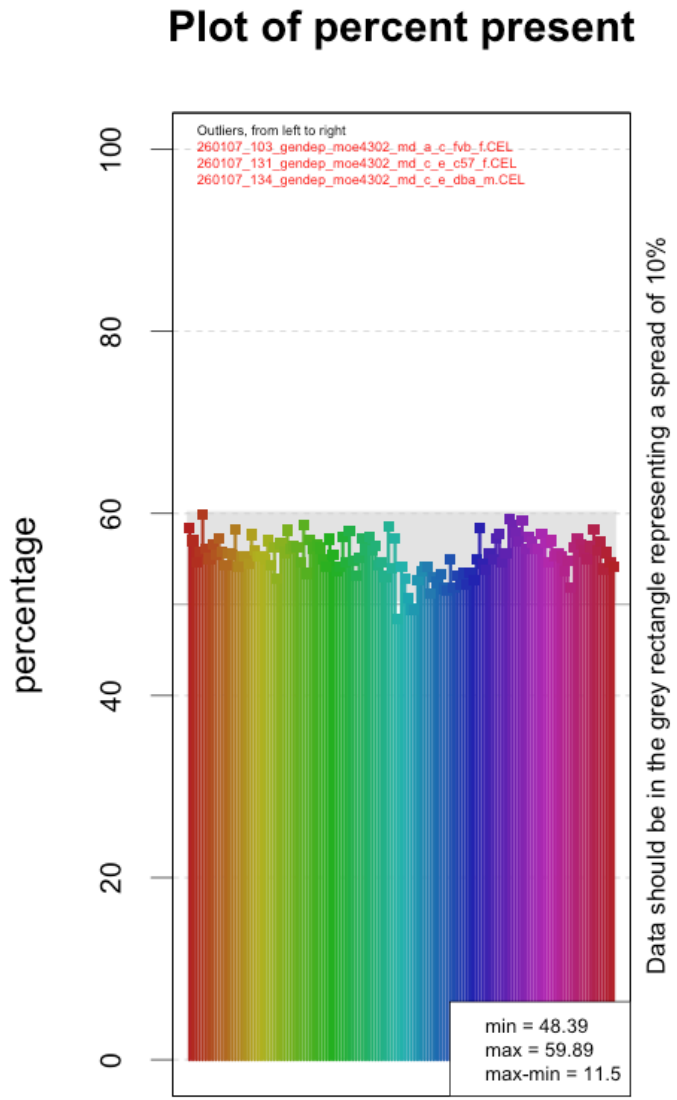

Plot of percent present
percentage
Outliers, from left to right
260107_103_gendep_moe4302_md_a_c_fvb_f.CEL
260107_131_gendep_moe4302_md_c_e_c57_f.CEL
260107_134_gendep_moe4302_md_c_e_dba_m.CEL
Data should be in the grey rectangle representing a spread of 10%
min = 48.39
max = 59.89
max-min = 11.5
0
20
40
60
80
100

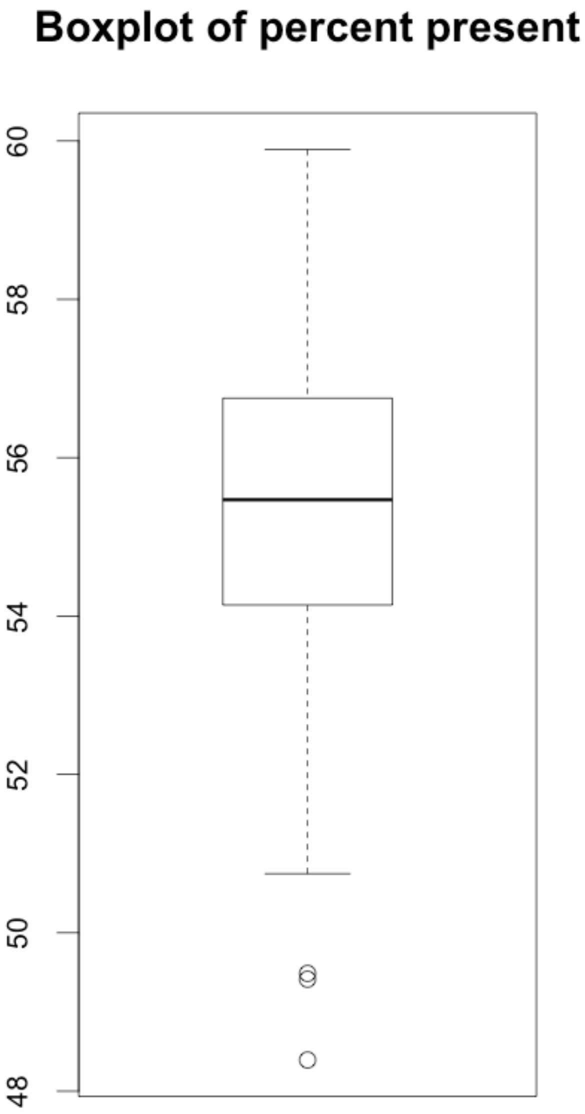

Boxplot of percent present
48
50
52
54
56
58
60

Relative Log Expression (RLE)

RLE distributions should be centered around 0

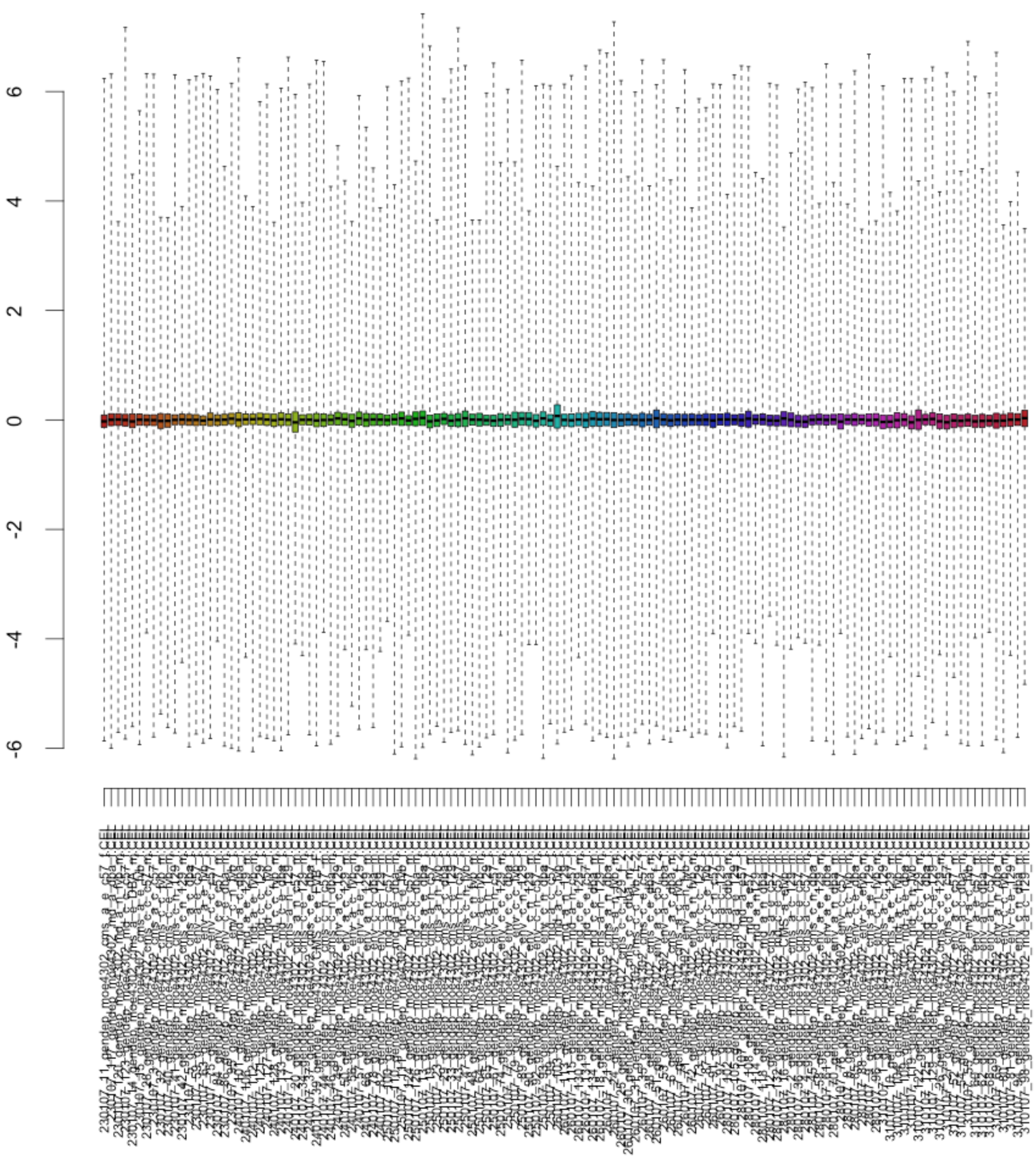

Supplement: Supplementary file 1 — Supporting Information. [file AJMG-174-235-s001.pdf]
