# Supplementary material for: Highly polygenic architecture of antidepressant treatment response: Comparative analysis of SSRI and NRI treatment in an animal model of depression
Source: Am J Med Genet B Neuropsychiatr Genet. 2016 Oct 1;174(3):235–50. doi: 10.1002/ajmg.b.32494 (PMC5434854; doi:10.1002/ajmg.b.32494)
Supplement: Supplementary file 2 — Supporting Information. [file AJMG-174-235-s002.pdf]

SSRI VS Control. List of probes uploaded to MetaCore for Pathway analysis.

Probe Set ID	Gene Symbol	Gene Title	Entrez Gene ID	Cytoband
1415709_s_at	Gbf1	golgi-specific brefeldin A-resistance factor 1	107338 Entrez gene	19 C3|19
1415753_at	Abhd17a	abhydrolase domain containing 17A	216169 Entrez gene	10 C1|10 39.72 cM
1415925_a_at	Nup62	nucleoporin 62	18226 Entrez gene	7 B4|7 23.1 cM
1416092_a_at	Map4	microtubule-associated protein 4	17758 Entrez gene	9 F2|9 59.83 cM
1416143_at	Atp5j	ATP synthase, H+ transporting, mitochondrial F0 complex, subunit F	11957 Entrez gene	16 C3.3|16
1416264_at	Abcb9	ATP-binding cassette, sub-family B (MDR/TAP), member 9	56325 Entrez gene	5 F|5
1416277_a_at	Gm13777<br>Rplp1	predicted gene 13777<br>ribosomal protein, large, P1	56040 Entrez gene<br>279067 Entrez gene	2 E1|2<br>9 B|9
1416392_a_at	Atp6v0c	ATPase, H+ transporting, lysosomal V0 subunit C	11984 Entrez gene	17 A3.3|17
1416990_at	Rxrb	retinoid X receptor beta	20182 Entrez gene	17 B1|17 17.98 cM
1416993_at	Cog4	component of oligomeric golgi complex 4	102339 Entrez gene	8 E1|8 57.76 cM
1417172_at	Ube2l6	ubiquitin-conjugating enzyme E2L 6	56791 Entrez gene	2|2 E1
1417326_a_at	Anapc11	anaphase promoting complex subunit 11	66156 Entrez gene	11 E2|11
1417807_at	Ufsp1	UFM1-specific peptidase 1	70240 Entrez gene	5 G2|5 76.33 cM
1417905_at	Prl7a2	prolactin family 7, subfamily a, member 2	19114 Entrez gene	13 A3.1|13 12.58 cM
1418027_at	Exo1	exonuclease 1	26909 Entrez gene	1 H4|1 81.9 cM
1418528_a_at	Dad1	defender against cell death 1	13135 Entrez gene	14 C2|14 27.7 cM
1418985_at	Cttnbp2nl	CTTNBP2 N-terminal like	80281 Entrez gene	3 F2.2|3
1419284_at	Cyhr1	cysteine and histidine rich 1	54151 Entrez gene	15|15 E1
1419673_at	Spock1	sparc/osteonectin, cwcv and kazal-like domains proteoglycan 1	20745 Entrez gene	13 B2|13
1419732_at	Rtn4r	reticulon 4 receptor	65079 Entrez gene	16 B1|16
1419755_at	Mfi2	antigen p97 (melanoma associated) identified by monoclonal antibodies 133.2 and 96.5	30060 Entrez gene	16|16 B3
1419857_at				
1419866_s_at	Atxn2	ataxin 2	20239 Entrez gene	5 F|5

1420192_at	Tmem191c	transmembrane protein 191C	224019 Entrez gene	16 A3|16 10.71 cM
1420641_a_at	Sqrdl	sulfide quinone reductase-like (yeast)	59010 Entrez gene	2|2 F2
1420749_a_at	Pou6f1	POU domain, class 6, transcription factor 1	19009 Entrez gene	15 F1|15 56.35 cM
1420938_at	Hs6st2	heparan sulfate 6-O-sulfotransferase 2	50786 Entrez gene	X|X A3.3
1421348_a_at	Cend1	cell cycle exit and neuronal differentiation 1	57754 Entrez gene	7 F5|7
1421891_at	St3gal2	ST3 beta-galactoside alpha-2,3-sialyltransferase 2	20444 Entrez gene	8 E1|8
1422119_at	Rab5b	RAB5B, member RAS oncogene family	19344 Entrez gene	10 D3|10
1422386_at	Olfr64	olfactory receptor 64	18366 Entrez gene	7 E3|7
1422480_at	Snx3	sorting nexin 3	54198 Entrez gene	10 B2|10 22.89 cM
1422492_at	Cpox	coproporphyrinogen oxidase	12892 Entrez gene	16 C1.1|16
1422506_a_at	Cstb	cystatin B	13014 Entrez gene	10 C1|10 39.72 cM
1423059_at	Ptk2	PTK2 protein tyrosine kinase 2	14083 Entrez gene	15 D3|15 33.94 cM
1423320_at	Dnase1l2	deoxyribonuclease 1-like 2	66705 Entrez gene	17 A3.3|17
1423344_at	Epor	erythropoietin receptor	13857 Entrez gene	9 A3|9 7.93 cM
1423389_at	Smad7	SMAD family member 7	17131 Entrez gene	18 E2|18
1423425_at	Plbd2	phospholipase B domain containing 2	71772 Entrez gene	5 F|5
1423560_at	Nell2	NEL-like 2	54003 Entrez gene	15 F1|15
1423647_a_at	Zdhhc3	zinc finger, DHHC domain containing 3	69035 Entrez gene	9 F4|9
1423785_at	Egln1	egl-9 family hypoxia-inducible factor 1	112405 Entrez gene	8 E2|8
1424106_at	Jkamp	JNK1/MAPK8-associated membrane protein	104771 Entrez gene	12 C3|12
1424384_a_at	Znrf1	zinc and ring finger 1	170737 Entrez gene	8 E1|8
1424476_at	Camkk2	calcium/calmodulin-dependent protein kinase kinase 2, beta	207565 Entrez gene	5 F|5
1424764_at	Sez6l	seizure related 6 homolog like	56747 Entrez gene	5 F|5
1425123_at	Klhl36	kelch-like 36	234796 Entrez gene	8 E1|8
1425233_at	2210407C18Rik	RIKEN cDNA 2210407C18 gene	78354	11 B1.3|11

			Entrez gene	
1425255_s_at	Hnrnpll	heterogeneous nuclear ribonucleoprotein L-like	72692 Entrez gene	17 E3|17
1425298_a_at	Naip1	NLR family, apoptosis inhibitory protein 1	17940 Entrez gene	13 D1-D3|13 53.18 cM
1425448_x_at	Atp6v0b	ATPase, H+ transporting, lysosomal V0 subunit B	114143 Entrez gene	4 D2.1|4
1425568_a_at	Tmem33	transmembrane protein 33	67878 Entrez gene	5|5 D
1425627_x_at	Gstm1	glutathione S-transferase, mu 1	14862 Entrez gene	3 F2.3|3
1425734_a_at	Ccdc77	coiled-coil domain containing 77	67200 Entrez gene	6 F1|6
1425860_x_at	Cklf	chemokine-like factor	75458 Entrez gene	8 D3|8
1426176_a_at	Prok2	prokineticin 2	50501 Entrez gene	6 D3|6 46.29 cM
1426508_at	Gfap	glial fibrillary acidic protein	14580 Entrez gene	11 D|11 66.48 cM
1426699_at	AU040320	expressed sequence AU040320	100317 Entrez gene	4 D2.2|4
1426894_s_at	Fam102a	family with sequence similarity 102, member A	98952 Entrez gene	2 B|2
1426959_at	Bdh1	3-hydroxybutyrate dehydrogenase, type 1	71911 Entrez gene	16 B2|16
1426989_at	Clstn3	calsyntenin 3	232370 Entrez gene	6 F2|6
1427286_at	Rbfox3	RNA binding protein, fox-1 homolog (C. elegans) 3	52897 Entrez gene	11 E2|11 83.22 cM
1427681_s_at	Vmn2r88	vomeronasal 2, receptor 88	669149 Entrez gene	14 C1|14
1428143_a_at	Pnpla2	patatin-like phospholipase domain containing 2	66853 Entrez gene	7 F5|7
1428619_at	Cox20	COX20 Cox2 chaperone	66359 Entrez gene	1|1 H3
1429113_at	Pagr1a<br>Prrt2	PAXIP1 associated glutamate rich protein 1A<br>proline-rich transmembrane protein 2	67278 Entrez gene<br>69017 Entrez gene	7 F3|7<br>7|7 F4
1429192_at	Ski	ski sarcoma viral oncogene homolog (avian)	20481 Entrez gene	4 E2|4 86.17 cM
1429490_at	Rif1	Rap1 interacting factor 1 homolog (yeast)	51869 Entrez gene	2 C1.1|2 29.98 cM
1429492_x_at	Ptdss2	phosphatidylserine synthase 2	27388 Entrez gene	7 F5|7
1429867_at	4933424C08Rik	RIKEN cDNA 4933424C08 gene	71185 Entrez gene	4|4
1429991_at	Fezf1	Fez family zinc finger 1	73191	6|6 A3

			Entrez gene	
1430246_at	4933406C10Rik	RIKEN cDNA 4933406C10 gene	74076 Entrez gene	12 A3|12
1430279_at	3110038A09Rik	RIKEN cDNA 3110038A09 gene	73126 Entrez gene	18|18
1431187_s_at	Dlg5	discs, large homolog 5 (Drosophila)	71228 Entrez gene	14 A3|14
1431752_a_at	Urm1	ubiquitin related modifier 1 homolog (S. cerevisiae)	68205 Entrez gene	2 B|2
1431849_at	Fam227a	family with sequence similarity 227, member A	75729 Entrez gene	15 E1|15
1431919_at	Rttn	rotatin	246102 Entrez gene	18 E4|18
1432268_at	2310068J16Rik	RIKEN cDNA 2310068J16 gene	70281 Entrez gene	15 F1|15
1432340_at	1700121N20Rik	RIKEN cDNA 1700121N20 gene	76639 Entrez gene	12 F1|12
1432463_at	5730510P18Rik	RIKEN cDNA 5730510P18 gene	70619 Entrez gene	12 A1.1|12
1432995_at	Panct1	pluripotency-associated noncoding transcript 1	73021 Entrez gene	X|X
1433380_at	4921509A06Rik	RIKEN cDNA 4921509A06 gene	70883 Entrez gene	9|9
1433889_at	Sox9	SRY (sex determining region Y)-box 9	20682 Entrez gene	11 E2|11 77.27 cM
1433928_a_at	Rpl13a	ribosomal protein L13A	22121 Entrez gene	7 B4|7 29.14 cM
1434359_at	Fam65b	family with sequence similarity 65, member B	193385 Entrez gene	13|13 A3.2
1434403_at	Spred2	sprouty-related, EVH1 domain containing 2	114716 Entrez gene	11|11 A3.2
1434560_at	Wdtc1	WD and tetratricopeptide repeats 1	230796 Entrez gene	4 D2.3|4
1434569_at	Tada2b	transcriptional adaptor 2B	231151 Entrez gene	5 B3|5
1434788_at	Fzd3	frizzled homolog 3 (Drosophila)	14365 Entrez gene	14 D1|14 34.09 cM
1434991_at	Fbxw17	F-box and WD-40 domain protein 17	109082 Entrez gene	13 A5|13
1435106_at	Limch1	LIM and calponin homology domains 1	77569 Entrez gene	5 C3.1|5
1435319_at	Ip6k2	inositol hexaphosphate kinase 2	76500 Entrez gene	9 F2|9
1435366_at	D430042O09Rik	RIKEN cDNA D430042O09 gene	233865 Entrez gene	7 F3|7
1435524_at	Snhg8	small nucleolar RNA host gene 8	69895 Entrez gene	3 G3|3
1435555_at	Pou2f1	POU domain, class 2, transcription factor 1	18986 Entrez gene	1 H2.3|1 73.21 cM

1435866_s_at	Hist3h2a	histone cluster 3, H2a	319162 Entrez gene	11 B2|11
1435961_at	Nat14	N-acetyltransferase 14	269854 Entrez gene	7 A1|7
1435986_x_at	Sdhc	succinate dehydrogenase complex, subunit C, integral membrane protein	66052 Entrez gene	1 H3|1
1436306_at	Ppp6r1	protein phosphatase 6, regulatory subunit 1	243819 Entrez gene	7 A1|7
1436622_at	Iqsec2	IQ motif and Sec7 domain 2	245666 Entrez gene	X F3|X 68.46 cM
1436720_s_at	Oog3	oogenesin 3	100012 Entrez gene	4 E1|4
1436747_at	Gm7367<br>Ubald2	1110014K08Rik pseudogene<br>UBA-like domain containing 2	319370 Entrez gene<br>664849 Entrez gene	11 E2|11<br>7 C|7
1436838_x_at	Cotl1	coactosin-like 1 (Dictyostelium)	72042 Entrez gene	8 E1|8
1436868_at	Rtn4rl1	reticulon 4 receptor-like 1	237847 Entrez gene	11 B5|11
1436924_x_at	Rpl31<br>Rpl31-ps12	ribosomal protein L31<br>ribosomal protein L31, pseudogene 1 2	114641 Entrez gene<br>665562 Entrez gene	1 B|1<br>16 A3|16
1437013_x_at	Atp6v0b	ATPase, H+ transporting, lysosomal V0 subunit B	114143 Entrez gene	4 D2.1|4
1437336_x_at	Prickle4<br>Tomm6	prickle homolog 4 (Drosophila)<br>translocase of outer mitochondrial membrane 6 homolog (yeast)	66119 Entrez gene<br>381104 Entrez gene	17 C|17
1437375_at	Rfx3	regulatory factor X, 3 (influences HLA class II expression)	19726 Entrez gene	19 C1|19
1437509_x_at	Prkd2	protein kinase D2	101540 Entrez gene	7 A2|7
1437633_at	Ankrd11	ankyrin repeat domain 11	77087 Entrez gene	8|8 E2
1437828_s_at	Wdr46	WD repeat domain 46	57315 Entrez gene	17 B1|17
1438054_x_at	Ppp2r2c	protein phosphatase 2, regulatory subunit B, gamma	269643 Entrez gene	5 B3|5
1438164_x_at	Flot2	flotillin 2	14252 Entrez gene	11 B5|11 46.74 cM
1438233_at	Fam178a	family with sequence similarity 178, member A	226151 Entrez gene	19 C3|19
1438338_at	Mdh1	malate dehydrogenase 1, NAD (soluble)	17449 Entrez gene	11 A3.1|11 13.89 cM
1438819_at	Nab1	Ngfi-A binding protein 1	17936 Entrez gene	1 C1.1|1 26.99 cM
1439232_at	Lhx1os	LIM homeobox 1, opposite strand	78365 Entrez gene	11|11

1439244_a_at	Tnrc6a	trinucleotide repeat containing 6a	233833 Entrez gene	7 F3|7
1439368_a_at	Slc9a3r2	solute carrier family 9 (sodium/hydrogen exchanger), member 3 regulator 2	65962 Entrez gene	17 A3.3|17
1439374_x_at	Rps10	ribosomal protein S10	67097 Entrez gene	17|17 B1
1439389_s_at	Myadm	myeloid-associated differentiation marker	50918 Entrez gene	7 A1|7 1.92 cM
1439924_x_at	Tubgcp5	tubulin, gamma complex associated protein 5	233276 Entrez gene	7 B5|7
1440064_at	Etl4	enhancer trap locus 4	208618 Entrez gene	2 A3|2 14.27 cM
1440239_at				
1440639_at	Dlgap1	discs, large (Drosophila) homolog-associated protein 1	224997 Entrez gene	17 E1.3|17 40.85 cM
1440867_at	Spry4	sprouty homolog 4 (Drosophila)	24066 Entrez gene	18 B3|18 20.5 cM
1441479_at				
1441859_x_at				
1441921_x_at	Esrrb	estrogen related receptor, beta	26380 Entrez gene	12 D2|12 40.49 cM
1441930_x_at	Vat1	vesicle amine transport protein 1 homolog (T californica)	26949 Entrez gene	11 D|11
1442195_at	BB283564	expressed sequence BB283564	554160 Entrez gene	
1442930_at	AW061147	expressed sequence AW061147	98662 Entrez gene	
1442963_at	Plcg1	phospholipase C, gamma 1	18803 Entrez gene	2 H2|2 80.97 cM
1442966_at				
1443776_at				
1444112_at	Gm15417	predicted gene 15417	545539 Entrez gene	3 F1|3
1444316_at				
1444606_at	Efna2	ephrin A2	13637 Entrez gene	10 C1|10 39.72 cM
1445763_at	1700013F07Rik	RIKEN cDNA 1700013F07 gene	75504 Entrez gene	3 F3|3
1446555_at				
1446712_at	Ntrk2	neurotrophic tyrosine kinase, receptor, type 2	18212 Entrez gene	13 B1-B2|13 31.2 cM
1447312_at				
1447725_at	C030034E14Rik	RIKEN cDNA C030034E14 gene	77469 Entrez gene	X|X
1447754_x_at	Thap4	THAP domain containing 4	67026 Entrez gene	1 D|1
1447916_at				

1448284_a_at	Ndufc1	NADH dehydrogenase (ubiquinone) 1, subcomplex unknown, 1	66377 Entrez gene	3|3 D
1449110_at	Rhob	ras homolog gene family, member B	11852 Entrez gene	12 A1.1|12
1449117_at	Jund	jun D proto-oncogene	16478 Entrez gene	8 C2|8 34.15 cM
1449118_at	Dbt	dihydrolipoamide branched chain transacylase E2	13171 Entrez gene	3 G1|3 50.37 cM
1449180_at	Kcmf1	potassium channel modulatory factor 1	74287 Entrez gene	6 C3|6 32.3 cM
1449445_x_at	Mfap1a<br>Mfap1b	microfibrillar-associated protein 1A<br>microfibrillar-associated protein 1B	67532 Entrez gene<br>100034361 Entrez gene	2 F1|2<br>2|2 F1
1449674_s_at	Pdcd6ip	programmed cell death 6 interacting protein	18571 Entrez gene	9|9 F2
1449942_a_at	Ilk	integrin linked kinase	16202 Entrez gene	7|7 E1
1450134_at	Loxl4	lysyl oxidase-like 4	67573 Entrez gene	19 D1|19
1450413_at	Pdgfb	platelet derived growth factor, B polypeptide	18591 Entrez gene	15 E|15 37.85 cM
1450436_s_at	Dnajb5	DnaJ (Hsp40) homolog, subfamily B, member 5	56323 Entrez gene	4|4 B1
1450797_a_at	Cbx1	chromobox 1	12412 Entrez gene	11 D|11 60.11 cM
1450925_a_at	Rps27l	ribosomal protein S27-like	67941 Entrez gene	9|9 D
1451098_at	Chmp1a	charged multivesicular body protein 1A	234852 Entrez gene	8 E1|8
1451110_at	Egln1	egl-9 family hypoxia-inducible factor 1	112405 Entrez gene	8 E2|8
1451574_at	Bcl9	B cell CLL/lymphoma 9	77578 Entrez gene	3 F2|3
1451869_at	Abca3	ATP-binding cassette, sub-family A (ABC1), member 3	27410 Entrez gene	17 A3.3|17
1452108_at	Igf1r	insulin-like growth factor I receptor	16001 Entrez gene	7 D1|7 37.27 cM
1452150_at	AU040320	expressed sequence AU040320	100317 Entrez gene	4 D2.2|4
1452485_at	Phospho1<br>Zfp652	phosphatase, orphan 1<br>zinc finger protein 652	237928 Entrez gene<br>268469 Entrez gene	11 D|11<br>11 D|11 59.01 cM
1452672_at	Thoc5	THO complex 5	107829 Entrez gene	11 A1|11
1452926_at	Ttc14	tetratricopeptide repeat domain 14	67120 Entrez gene	3|3 B
1452965_at	Ankrd13d	ankyrin repeat domain 13 family, member D	68423 Entrez gene	19 A|19

1453111_a_at	Slc25a39	solute carrier family 25, member 39	68066 Entrez gene	11 D|11 66.29 cM
1453118_s_at	Rpl22	ribosomal protein L22	19934 Entrez gene	4 E2|4
1453494_at	4921513H07Rik	RIKEN cDNA 4921513H07 gene	100504457 Entrez gene	6 F3|6
1453729_a_at	Gm13826<br>Rpl37	predicted gene 13826<br>ribosomal protein L37	67281 Entrez gene 100502825 Entrez gene	15 A1|15 5|5 55.99 cM
1453819_x_at	Stx18	syntaxin 18	71116 Entrez gene	5 B3|5
1454160_at	Grip1os1	glutamate receptor interacting protein 1, opposite strand 1	78105 Entrez gene	10|10
1454346_at	4930540E01Rik	RIKEN cDNA 4930540E01 gene	75174 Entrez gene	9|9
1454721_at	Arel1	apoptosis resistant E3 ubiquitin protein ligase 1	68497 Entrez gene	12 D2|12
1454729_at	Tmem108	transmembrane protein 108	81907 Entrez gene	9 F1|9
1454808_at	Micu2	mitochondrial calcium uptake 2	68514 Entrez gene	14 C2|14
1455025_at	Paqr9	progestin and adipoQ receptor family member IX	75552 Entrez gene	9 E3.3|9
1455138_x_at	Cfl1	cofilin 1, non-muscle	12631 Entrez gene	19 A|19
1455158_at	Itga3	integrin alpha 3	16400 Entrez gene	11 D|11 59.01 cM
1455713_x_at	Phb2	prohibitin 2	12034 Entrez gene	6 F2|6 59.17 cM
1455826_a_at	Bace1	beta-site APP cleaving enzyme 1	23821 Entrez gene	9 A5.2|9
1456180_at	Rbm24	RNA binding motif protein 24	666794 Entrez gene	13 A5|13
1456373_x_at	Rps20	ribosomal protein S20	67427 Entrez gene	4 A1|4
1456872_a_at	Harbi1	harbinger transposase derived 1	241547 Entrez gene	2 E1|2
1457335_at	Gm16794	predicted gene, 16794	100504734 Entrez gene	9|9 50.3 cM
1457946_at	Sebox	SEBOX homeobox	18292 Entrez gene	11 B5|11 46.74 cM
1458105_at	Syn2	synapsin II	20965 Entrez gene	6 F|6 53.2 cM
1458164_at	Gm14634	predicted gene 14634	552913 Entrez gene	X|X 7.61 cM
1458538_at				
1458854_at	C78891	expressed sequence C78891	97714 Entrez gene	

1459413_at				
1459998_at	Zfp407	zinc finger protein 407	240476<br>Entrez gene	18 E4|18
1460003_at	AI956758	expressed sequence AI956758	99132<br>Entrez gene	
1460008_x_at	Rpl31<br>Rpl31-ps12	ribosomal protein L31<br>ribosomal protein L31, pseudogene 1 2	114641<br>Entrez gene<br>665562<br>Entrez gene	1 B|1<br>16 A3|16
1460034_at	Samd4b	sterile alpha motif domain containing 4B	233033<br>Entrez gene	7 A3|7
1460048_at				
1460172_at	Cdip1	cell death inducing Trp53 target 1	66626<br>Entrez gene	16 A1|16<br>2.47 cM
1460405_at	Arhgef10l	Rho guanine nucleotide exchange factor (GEF) 10-like	72754<br>Entrez gene	4 D3|4
1460646_at	Csnk2a2	casein kinase 2, alpha prime polypeptide	13000<br>Entrez gene	8 D1|8<br>47.12 cM
1460654_at	Slc30a3	solute carrier family 30 (zinc transporter), member 3	22784<br>Entrez gene	5 B1|5<br>16.97 cM
1460678_at	Klhdc2	kelch domain containing 2	69554<br>Entrez gene	12 C3|12<br>28.77 cM
1460724_at	Ap2a1	adaptor-related protein complex 2, alpha 1 subunit	11771<br>Entrez gene	7|7 B2

NRI vs Saline. List of probes uploaded to MetaCore for pathway analysis .

Probe Set ID	Gene Symbol	Gene Title	Entrez Gene ID	Cytoband
1415734_at	Rab7	RAB7, member RAS oncogene family	19349 Entrez gene	6 D1|6 39.13 cM
1415907_at	Ccnd3	cyclin D3	12445 Entrez gene	17 C|17 23.37 cM
1415909_at	Stip1	stress-induced phosphoprotein 1	20867 Entrez gene	19 A|19
1416018_at	Dr1	down-regulator of transcription 1	13486 Entrez gene	5 F|5 52.82 cM
1416097_at	Lrrc4	leucine rich repeat containing 4	192198 Entrez gene	6 A3.3|6
1416173_at	Pes1	pescadillo homolog 1, containing BRCT domain (zebrafish)	64934 Entrez gene	11 A1|11
1416220_at	Spcs1	signal peptidase complex subunit 1 homolog (S. cerevisiae)	69019 Entrez gene	14 B|14
1416350_at	Klf16	Kruppel-like factor 16	118445 Entrez gene	10 C1|10
1416465_a_at	Vapa	vesicle-associated membrane protein, associated protein A	30960 Entrez gene	17|17 E1.2
1416610_a_at	Clcn3	chloride channel 3	12725 Entrez gene	8 B3.1|8 30.9 cM
1416666_at	Serpine2	serine (or cysteine) peptidase inhibitor, clade E, member 2	20720 Entrez gene	1 C4|1 40.97 cM
1416796_at	Nck2	non-catalytic region of tyrosine kinase adaptor protein 2	17974 Entrez gene	1|1 C1
1416876_at	Parvg	parvin, gamma	64099 Entrez gene	15 E2|15
1416918_at	Dlg3	discs, large homolog 3 (Drosophila)	53310 Entrez gene	X|X C2
1417449_at	Acot8	acyl-CoA thioesterase 8	170789 Entrez gene	2 H3|2
1417479_at	Ppp2r3c	protein phosphatase 2, regulatory subunit B", gamma	59032 Entrez gene	12 C1|12
1417606_a_at	Calr	calreticulin	12317	8 C3|8

			Entrez gene	41.21 cM
1417632_at	Atp6v0a1	ATPase, H+ transporting, lysosomal V0 subunit A1	11975 Entrez gene	11 D|11 64.04 cM
1417749_a_at	Tjp1	tight junction protein 1	21872 Entrez gene	7 C|7 35.02 cM
1418265_s_at	Irf2	interferon regulatory factor 2	16363 Entrez gene	8|8 B2
1418524_at	Pcm1	pericentriolar material 1	18536 Entrez gene	8 A4|8 23.89 cM
1418789_at	Sntg2	syntrophin, gamma 2	268534 Entrez gene	12|12 B1
1419550_a_at	Stk39	serine/threonine kinase 39	53416 Entrez gene	2|2 C3
1419719_at	Gabrb1	gamma-aminobutyric acid (GABA) A receptor, subunit beta 1	14400 Entrez gene	5 C3.2|5 38.18 cM
1419821_s_at	Idh1	isocitrate dehydrogenase 1 (NADP+), soluble	15926 Entrez gene	1 C2|1 32.91 cM
1419831_at	AA416453	expressed sequence AA416453	99996 Entrez gene	
1420013_s_at	Lss	lanosterol synthase	16987 Entrez gene	10 C1|10 39.1 cM
1420019_at	Tspan8	tetraspanin 8	216350 Entrez gene	10 D2|10
1420368_at	Denr	density-regulated protein	68184 Entrez gene	5 F|5
1420836_at	Slc25a30	solute carrier family 25, member 30	67554 Entrez gene	14|14 D2
1420957_at	Apc	adenomatosis polyposis coli	11789 Entrez gene	18 B1|18 18.53 cM
1421055_at	Lats2 Xpo4	large tumor suppressor 2 exportin 4	50523 Entrez gene 57258 Entrez gene	14 C3|14
1421516_at	Nr6a1	nuclear receptor subfamily 6, group A, member 1	14536 Entrez	2 B|2

			gene	
1421748_a_at	Tubd1	tubulin, delta 1	56427 Entrez gene	11 C|11
1422117_s_at	Khdrbs2	KH domain containing, RNA binding, signal transduction associated 2	170771 Entrez gene	1 B|1
1422511_a_at	Ogfr	opioid growth factor receptor	72075 Entrez gene	2 H4|2
1423375_at	Cir1	corepressor interacting with RBPJ, 1	66935 Entrez gene	2 C3|2
1423389_at	Smad7	SMAD family member 7	17131 Entrez gene	18 E2|18
1423413_at	Ndrg1	N-myc downstream regulated gene 1	17988 Entrez gene	15 D2|15
1423562_at	Prrt1	proline-rich transmembrane protein 1	260297 Entrez gene	17 B1|17
1423922_s_at	Ints3	integrator complex subunit 3	229543 Entrez gene	3 F1|3
1424106_at	Jkamp	JNK1/MAPK8-associated membrane protein	104771 Entrez gene	12 C3|12
1424399_at	Uck1	uridine-cytidine kinase 1	22245 Entrez gene	2 B|2
1424416_at	Nkiras2	NFKB inhibitor interacting Ras-like protein 2	71966 Entrez gene	11 D|11
1424683_at	Fam134b	family with sequence similarity 134, member B	66270 Entrez gene	15 B1|15
1425204_s_at	Ddx19a Ddx19b	DEAD (Asp-Glu-Ala-Asp) box polypeptide 19a DEAD (Asp-Glu-Ala-Asp) box polypeptide 19b	13680 Entrez gene 234733 Entrez gene	8 E1|8 8|8 D3
1425281_a_at	Tsc22d3	TSC22 domain family, member 3	14605 Entrez gene	X F1|X
1425482_s_at	Ankmy2	ankyrin repeat and MYND domain containing 2	217473 Entrez gene	12 A3|12
1425690_at	B3gat1	beta-1,3-glucuronyltransferase 1 (glucuronosyltransferase P)	76898 Entrez gene	9 A4|9

1426315_a_at	6330416G13Rik	RIKEN cDNA 6330416G13 gene	230279 Entrez gene	4 C1|4
1426640_s_at	Trib2	tribbles homolog 2 (Drosophila)	217410 Entrez gene	12 A1.1|12
1426699_at	AU040320	expressed sequence AU040320	100317 Entrez gene	4 D2.2|4
1426959_at	Bdh1	3-hydroxybutyrate dehydrogenase, type 1	71911 Entrez gene	16 B2|16
1427385_s_at	Actn1	actinin, alpha 1	109711 Entrez gene	12 C3|12 36.49 cM
1428017_at	Pknox2	Pbx/knotted 1 homeobox 2	208076 Entrez gene	9 A4|9
1428107_at	Sh3bgrl	SH3-binding domain glutamic acid-rich protein like	56726 Entrez gene	X D|X
1428337_at	Mdp1	magnesium-dependent phosphatase 1	67881 Entrez gene	14 C3|14
1428962_at	1700013F07Rik	RIKEN cDNA 1700013F07 gene	75504 Entrez gene	3 F3|3
1429593_at	Slc38a2	solute carrier family 38, member 2	67760 Entrez gene	15 F1|15
1429879_at	0610037L13Rik	RIKEN cDNA 0610037L13 gene	74098 Entrez gene	4 C7|4
1429899_at	5730414N17Rik	RIKEN cDNA 5730414N17 gene	70524 Entrez gene	
1431182_at	Hspa8	heat shock protein 8	15481 Entrez gene	9 A5.1|9 21.55 cM
1431412_at	2810455B08Rik	RIKEN cDNA 2810455B08 gene	72777 Entrez gene	16|16
1431443_at	1810008B01Rik	RIKEN cDNA 1810008B01 gene	69062 Entrez gene	8|8
1431795_a_at	Sema3b	sema domain, immunoglobulin domain (Ig), short basic domain, secreted, (semaphorin) 3B	20347 Entrez gene	9 F1|9 58.31 cM
1432400_at	Epha1	Eph receptor A1	13835 Entrez gene	6 B2.1|6

1432508_at	Ptges3l	prostaglandin E synthase 3 (cytosolic)-like	73635 Entrez gene	11 D|11
1432754_at	Ccnc	cyclin C	51813 Entrez gene	4 A3|4
1432826_a_at	Cd80	CD80 antigen	12519 Entrez gene	16 B5|16 26.86 cM
1432995_at	Panct1	pluripotency-associated noncoding transcript 1	73021 Entrez gene	X|X
1433356_at	2610021J01Rik	RIKEN cDNA 2610021J01 gene	69214 Entrez gene	
1433806_x_at	Calr	calreticulin	12317 Entrez gene	8 C3|8 41.21 cM
1433889_at	Sox9	SRY (sex determining region Y)-box 9	20682 Entrez gene	11 E2|11 77.27 cM
1434133_s_at	Dcaf8	DDB1 and CUL4 associated factor 8	98193 Entrez gene	1 H3|1 79.54 cM
1434151_at	Mettl7a1	methyltransferase like 7A1	70152 Entrez gene	15|15 F3
1434222_at	Sipa1l1	signal-induced proliferation-associated 1 like 1	217692 Entrez gene	12 D1|12
1434325_x_at	Prkar1b	protein kinase, cAMP dependent regulatory, type I beta	19085 Entrez gene	5 G2|5 77.74 cM
1434514_at	Rbm15	RNA binding motif protein 15	229700 Entrez gene	3 F2.3|3
1434760_at	Lrrtm3	leucine rich repeat transmembrane neuronal 3	216028 Entrez gene	10 B4|10
1434982_at	Rnf182	ring finger protein 182	328234 Entrez gene	13 A4|13
1434985_a_at	Eif4a1	eukaryotic translation initiation factor 4A1	13681 Entrez gene	11 B3|11 42.86 cM
1434995_s_at	Dedd	death effector domain-containing	21945 Entrez gene	1 H2|1 79.34 cM
1435106_at	Limch1	LIM and calponin homology domains 1	77569 Entrez gene	5 C3.1|5
1435219_x_at	Becn1	beclin 1, autophagy related	56208	11 D|11

			Entrez gene	
1435303_at	Taf4b	TAF4B RNA polymerase II, TATA box binding protein (TBP)-associated factor	72504 Entrez gene	18 A1|18
1435434_at	Braf	Braf transforming gene	109880 Entrez gene	6 B1|6 18.43 cM
1435435_at	Cttnbp2	cortactin binding protein 2	30785 Entrez gene	6 A2|6
1435626_a_at	Herpud1	homocysteine-inducible, endoplasmic reticulum stress-inducible, ubiquitin-like domain member 1	64209 Entrez gene	8 C5|8
1435839_at	Gm7694	predicted gene 7694	665574 Entrez gene	1 H3|1
1435962_at	LOC102643254 Rps6 Rps6-ps4	40S ribosomal protein S6-like ribosomal protein S6 ribosomal protein S6, pseudogene 4	20104 Entrez gene 667739 Entrez gene 102643254 Entrez gene	2 C3|2 4|4 C3
1436302_at	Slc10a7	solute carrier family 10 (sodium/bile acid cotransporter family), member 7	76775 Entrez gene	8|8 C3
1436428_at	Chrnb2	cholinergic receptor, nicotinic, beta polypeptide 2 (neuronal)	11444 Entrez gene	3 F1|3 39.19 cM
1436637_at				
1436720_s_at	Oog3	oogenesin 3	100012 Entrez gene	4 E1|4
1436953_at	Wipf1	WAS/WASL interacting protein family, member 1	215280 Entrez gene	2 C3|2 43.68 cM
1437378_x_at	Scarb1	scavenger receptor class B, member 1	20778 Entrez gene	5 G1.1|5 64.11 cM
1437391_x_at	Mrpl44	mitochondrial ribosomal protein L44	69163 Entrez gene	1 C4|1
1437490_x_at	Uap1	UDP-N-acetylglucosamine pyrophosphorylase 1	107652 Entrez gene	1 H3|1
1437546_at	Dnajc14	DnaJ (Hsp40) homolog, subfamily C, member 14	74330 Entrez gene	10 D3|10
1437775_at	Dlst	dihydrolipoamide S-succinyltransferase (E2	78920	12|12 D3

		component of 2-oxo-glutarate complex)	Entrez gene	
1437908_a_at	Ergic1	endoplasmic reticulum-golgi intermediate compartment (ERGIC) 1	67458 Entrez gene	17|17 B1
1438064_at	Ybx1	Y box protein 1	22608 Entrez gene	4|4 D1
1438104_at				
1438105_at	March10	membrane-associated ring finger (C3HC4) 10	632687 Entrez gene	11 E1|11
1438116_x_at	Slc9a3r1	solute carrier family 9 (sodium/hydrogen exchanger), member 3 regulator 1	26941 Entrez gene	11 E2|11
1438244_at	Nfib	nuclear factor I/B	18028 Entrez gene	4 C4-C6|4 38.4 cM
1438313_at				
1438357_at	Pfdn5	prefoldin 5	56612 Entrez gene	15 F3|15 57.48 cM
1438360_x_at	Slc25a5	solute carrier family 25 (mitochondrial carrier, adenine nucleotide translocator), member 5	11740 Entrez gene	X A4|X 21.2 cM
1438380_at	Ddx47	DEAD (Asp-Glu-Ala-Asp) box polypeptide 47	67755 Entrez gene	6 G1|6
1438530_at	Tfpi	tissue factor pathway inhibitor	21788 Entrez gene	2 D|2
1438819_at	Nab1	Ngfi-A binding protein 1	17936 Entrez gene	1 C1.1|1 26.99 cM
1438920_x_at	Cr1l	complement component (3b/4b) receptor 1-like	12946 Entrez gene	1 H6|1 98.43 cM
1438925_x_at	Atp6v0c	ATPase, H+ transporting, lysosomal V0 subunit C	11984 Entrez gene	17 A3.3|17
1438930_s_at	Mecp2	methyl CpG binding protein 2	17257 Entrez gene	X A7.3|X 37.63 cM
1439124_at	Wdr91	WD repeat domain 91	101240 Entrez gene	6 B1|6
1439364_a_at	Mmp2	matrix metallopeptidase 2	17390 Entrez gene	8 C5|8 44.99 cM
1439385_x_at	Slc13a3	solute carrier family 13 (sodium-dependent dicarboxylate transporter), member 3	114644 Entrez	2 H3|2

			gene	
1439597_at				
1440013_at				
1440055_at				
1440064_at	Etl4	enhancer trap locus 4	208618 Entrez gene	2 A3l2 14.27 cM
1440459_at	Setx	senataxin	269254 Entrez gene	2 A3l2
1440560_at				
1440749_at				
1440997_at	Tnrc6c	trinucleotide repeat containing 6C	217351 Entrez gene	11 E2l11
1441155_at				
1441348_at	Zfp955a	zinc finger protein 955A	77652 Entrez gene	17 B1l17
1441349_at	LOC552904	uncharacterized LOC552904	552904 Entrez gene	
1441479_at				
1441676_at	Zfat	zinc finger and AT hook domain containing	380993 Entrez gene	15 D2l15
1441921_x_at	Esrrb	estrogen related receptor, beta	26380 Entrez gene	12 D2l12 40.49 cM
1441928_x_at	Ell	elongation factor RNA polymerase II	13716 Entrez gene	8 C1l8
1442087_at	H3f3a	H3 histone, family 3A	15078 Entrez gene	1l1 D2.3
1442409_at	D9Wsu90e	DNA segment, Chr 9, Wayne State University 90, expressed	27962 Entrez gene	9 43.38 cM
1442995_at				
1443005_at	Zeb1	zinc finger E-box binding homeobox 1	21417 Entrez gene	18 A1l18 4.42 cM
1443074_at				
1443247_at				
1443393_at				
1443551_at	Atp2a2	ATPase, Ca++ transporting, cardiac muscle, slow twitch 2	11938 Entrez gene	5 F l5 62.38 cM
1443566_at				

1443836_x_at	Wdr48	WD repeat domain 48	67561 Entrez gene	9 F4|9
1444273_at	AW555355	expressed sequence AW555355	99413 Entrez gene	
1444316_at				
1444436_at	9630030I15Rik Hsf2	RIKEN cDNA 9630030I15 gene heat shock factor 2	15500 Entrez gene 319253 Entrez gene	10|10 10|10 B3-B4
1444519_at	Lgr5	leucine rich repeat containing G protein coupled receptor 5	14160 Entrez gene	10 D2|10
1444604_at				
1444745_at				
1445066_at				
1445167_at	9630001P10Rik	RIKEN cDNA 9630001P10 gene	319251 Entrez gene	5 B3|5
1445391_at	Diap1	diaphanous homolog 1 (Drosophila)	13367 Entrez gene	18 B3|18 19.71 cM
1445649_x_at	Zfp142	zinc finger protein 142	77264 Entrez gene	1 C3|1 38.54 cM
1446185_at	Mtor	mechanistic target of rapamycin (serine/threonine kinase)	56717 Entrez gene	4 E1|4 78.76 cM
1446260_at	4930447C04Rik	RIKEN cDNA 4930447C04 gene	75801 Entrez gene	12 C3|12
1446311_at	Esyt1	extended synaptotagmin-like protein 1	23943 Entrez gene	10 D3|10
1446340_at				
1446608_at				
1446811_at				
1446949_at	4930431P19Rik	RIKEN cDNA 4930431P19 gene	73886 Entrez gene	7 E3|7
1447099_at				
1447114_x_at	4930414L22Rik	RIKEN cDNA 4930414L22 gene	78108 Entrez gene	6|6
1447188_at				
1447462_at				
1447653_x_at	Gm9385	predicted pseudogene 9385	68193	16 C1|16

	Rpl24	ribosomal protein L24	Entrez gene 668829 Entrez gene	33.74 cM 9 F3|9
1447951_at	AK153988	Mus musculus 0 day neonate skin cDNA, RIKEN full-length enriched library, clone:4631401N08 product:hypothetical protein, full insert sequence.		
1448114_a_at	Trmt2a	TRM2 tRNA methyltransferase 2A	15547 Entrez gene	16 A3|16 11.31 cM
1448306_at	Nfkbia	nuclear factor of kappa light polypeptide gene enhancer in B cells inhibitor, alpha	18035 Entrez gene	12|12 C1-C3
1448324_at	Rnps1	ribonucleic acid binding protein S1	19826 Entrez gene	17 A3.3|17
1448985_at	Dusp22	dual specificity phosphatase 22	105352 Entrez gene	13 A3.2|13
1448996_at	Rom1	rod outer segment membrane protein 1	19881 Entrez gene	19 A|19 6.03 cM
1449457_at	Acot12	acyl-CoA thioesterase 12	74156 Entrez gene	13 C3|13
1449572_at	Trhr	thyrotropin releasing hormone receptor	22045 Entrez gene	15 B3.2|15 16.82 cM
1449584_at	Dgkg	diacylglycerol kinase, gamma	110197 Entrez gene	16 B1|16 13.37 cM
1449674_s_at	Pdcd6ip	programmed cell death 6 interacting protein	18571 Entrez gene	9|9 F2
1450350_a_at	Jdp2	Jun dimerization protein 2	81703 Entrez gene	12|12 D3
1450472_s_at	Smad3	SMAD family member 3	17127 Entrez gene	9 D|9
1450853_at	Tle4	transducin-like enhancer of split 4, homolog of Drosophila E(spl)	21888 Entrez gene	19 A|19 9.11 cM
1450983_at	Akap8	A kinase (PRKA) anchor protein 8	56399 Entrez gene	17|17 B2
1451046_at	Zfpm1	zinc finger protein, multitype 1	22761 Entrez gene	8 E1|8
1451251_at	Appbp2	amyloid beta precursor protein (cytoplasmic tail)	66884	11|11 B5

		binding protein 2	Entrez gene	
1451353_at	Tm6sf1	transmembrane 6 superfamily member 1	107769 Entrez gene	7 D1l7
1452144_a_at	Mrpl44	mitochondrial ribosomal protein L44	69163 Entrez gene	1 C4l1
1452150_at	AU040320	expressed sequence AU040320	100317 Entrez gene	4 D2.2l4
1452254_at	Mtmr9	myotubularin related protein 9	210376 Entrez gene	14 D1l14
1452657_at	Ap1s2	adaptor-related protein complex 1, sigma 2 subunit	108012 Entrez gene	X F5lX
1452724_at	Ppp1r16a	protein phosphatase 1, regulatory (inhibitor) subunit 16A	73062 Entrez gene	15 D3l15 36.25 cM
1452769_at	Rnf145	ring finger protein 145	74315 Entrez gene	11 B1.1l11
1453336_at	Fam175a	family with sequence similarity 175, member A	70681 Entrez gene	5 E4l5
1453720_at	Rnf157	ring finger protein 157	217340 Entrez gene	11 E2l11
1453846_at	Chpf2	chondroitin polymerizing factor 2	100910 Entrez gene	5 A3l5
1454055_at	1600029O15Rik	ribosomal protein L17 pseudogene	665268 Entrez gene	9 Bl9
1454571_at	A930036A04Rik	RIKEN cDNA A930036A04 gene	77839 Entrez gene	12l12
1454593_at	Fgd3	FYVE, RhoGEF and PH domain containing 3	30938 Entrez gene	13 A5l13 25.18 cM
1454787_at	Zdhhc9	zinc finger, DHHC domain containing 9	208884 Entrez gene	X A4lX
1455421_x_at	Fam131b	family with sequence similarity 131, member B	76156 Entrez gene	6 B2l6
1455470_x_at	Lasp1	LIM and SH3 protein 1	16796 Entrez gene	11 C-Dl11 61.1 cM
1455660_at	Csf2rb	colony stimulating factor 2 receptor, beta, low-affinity (granulocyte-macrophage)	12983 Entrez	15 E1l15 37.36 cM

			gene	
1455713_x_at	Phb2	prohibitin 2	12034 Entrez gene	6 F2l6 59.17 cM
1455826_a_at	Bace1	beta-site APP cleaving enzyme 1	23821 Entrez gene	9 A5.2l9
1456048_at	Cpeb3	cytoplasmic polyadenylation element binding protein 3	208922 Entrez gene	19 C2l19
1456099_at	D930017J03Rik	RIKEN cDNA D930017J03 gene	320391 Entrez gene	14l14
1456106_x_at	Sdccag3	serologically defined colon cancer antigen 3	68112 Entrez gene	2 A3l2
1456110_at	Ankrd11	ankyrin repeat domain 11	77087 Entrez gene	8l8 E2
1456226_x_at	Ddr1	discoidin domain receptor family, member 1	12305 Entrez gene	17 Cl17 18.7 cM
1456304_at	Gm996	predicted gene 996	381353 Entrez gene	2 A3l2
1456394_at	Rint1	RAD50 interactor 1	72772 Entrez gene	5 A3l5
1456516_x_at	Uap1	UDP-N-acetylglucosamine pyrophosphorylase 1	107652 Entrez gene	1 H3l1
1456701_at	Rabl6	RAB, member RAS oncogene family-like 6	227624 Entrez gene	2 A3l2
1456755_at	Trak1	trafficking protein, kinesin binding 1	67095 Entrez gene	9 F4l9 72.41 cM
1456779_a_at	4930414L22Rik	RIKEN cDNA 4930414L22 gene	78108 Entrez gene	6l6
1456855_at				
1456985_at	Dnttip1	deoxynucleotidyltransferase, terminal, interacting protein 1	76233 Entrez gene	2 H3l2
1457543_at	Nsun7	NOL1/NOP2/Sun domain family, member 7	70918 Entrez gene	5l5 D
1457635_s_at	Nr3c1	nuclear receptor subfamily 3, group C, member 1	14815 Entrez gene	18 B3l18 21.09 cM
1457662_x_at	Tpk1	thiamine pyrophosphokinase	29807	6l6 B2

			Entrez gene	
1457864_at	Rab11fip3	RAB11 family interacting protein 3 (class II)	215445 Entrez gene	17 A3.3|17
1457946_at	Sebox	SEBOX homeobox	18292 Entrez gene	11 B5|11 46.74 cM
1458116_at	Spaca6	sperm acrosome associated 6	75202 Entrez gene	17 A3.2|17
1458190_at	Arhgap4	Rho GTPase activating protein 4	171207 Entrez gene	X A7.3|X
1458322_x_at	E230008N13Rik	RIKEN cDNA E230008N13 gene	381522 Entrez gene	4 B1|4
1458383_at				
1458439_a_at	Dzip3	DAZ interacting protein 3, zinc finger	224170 Entrez gene	16 B5|16
1458538_at				
1458609_at	Gm20161	predicted gene, 20161	100504304 Entrez gene	17|17 15.46 cM
1458715_at	Casp6	caspase 6	12368 Entrez gene	3|3 H1
1458729_at				
1459188_at				
1459403_at	Ldlr	low density lipoprotein receptor	16835 Entrez gene	9 A3|9 7.87 cM
1459411_at				
1459567_at	C77905	expressed sequence C77905	97142 Entrez gene	
1459862_at				
1459909_at	Nfix	nuclear factor I/X	18032 Entrez gene	8 C1-C2|8 41.02 cM
1459941_at	Clvs1	clavesin 1	74438 Entrez gene	4 A1|4
1459998_at	Zfp407	zinc finger protein 407	240476 Entrez gene	18 E4|18
1460055_at				
1460163_at	C1qtnf7	C1q and tumor necrosis factor related protein 7	109323 Entrez	5 B3|5

			gene	
1460357_at	Ythdf2	YTH domain family 2	213541 Entrez gene	4 D2.3l4
1460619_at	Mfsd9	major facilitator superfamily domain containing 9	211798 Entrez gene	1 Bl1
1460650_at	Atp6v0a1	ATPase, H+ transporting, lysosomal V0 subunit A1	11975 Entrez gene	11 Dl11 64.04 cM
1460679_at	Exosc4	exosome component 4	109075 Entrez gene	15 D3l15
