# Supplementary material for: Highly polygenic architecture of antidepressant treatment response: Comparative analysis of SSRI and NRI treatment in an animal model of depression
Source: Am J Med Genet B Neuropsychiatr Genet. 2016 Oct 1;174(3):235–50. doi: 10.1002/ajmg.b.32494 (PMC5434854; doi:10.1002/ajmg.b.32494)
Supplement: Supplementary file 3 — Supporting Information. [file AJMG-174-235-s003.pdf]

Supplementary information

Information Gain

The Information Theory-based filter ”Information Gain” (InfoGain) assesses each feature importance according to its mutual information with the label. That is $H(X_j, Y)$ is computed for every feature X_j and a ranking of the features is produced by sorting the features increasingly according to this measure.

mrmr

This filter is also based on mutual information measures. However, its computation is more complex and time consuming. It belongs to a class of feature selection techniques known as forward greedy selection, which general functioning can be seen in Algorithm 1.

Algorithm 1 Forward greedy selection

```
Input: k                              Number of features to select.
  S = {}                              Ordered set
  D = {All features}
  while |S| < k do
    X* ← best feature in D
    S ← S ∪ {X*}
    D ← D \ {X*}
  end while
  return S
```

For the case of mrmr, in each iteration the features are assessed according to

$$J(X_i) = H(X_i, Y) - \frac{1}{|S|} \sum_{j \in S} H(X_i, X_j). \tag{1}$$

The first term represents the relevance of the feature and it is equivalent to the assessment of Information Gain. The second term tries to account for the redundancy of a feature with the ones that have already been selected, thus avoiding to select features that do not add extra information.

It is worth noting that mrmr requires a significantly higher number of calculations and is computationally costly.

We have explored some Information Theory-based filters that work by computing mutual informations. This measure can be computed for both continuous and discrete variables. However, the computation for the continuous case requires computing integrals, therefore density estimation techniques are required. In this case, we choose to discretise the variables in order to compute mutual informations.

One-way ANOVA

The one-way ANOVA test is used to check if the means of several groups are statistically different. It does so by computing the so-called F-score and extracting a p-value. In this study, the F-scores of each feature was computed and used to rank the variables.

In order to compute the F-score, this test relies on the following assumptions:

- The samples are independent.
- The population is normally distributed.
- Each group has the same variance.

This assumptions have to be true in order for the test to be relevant. However, one-way ANOVA has shown to be robust even when these assumptions are lightly violated.

The F-score is computed as a ratio between the between groups variance and the withing group variances. The between group variance is computed as

$$s_{\text{between}} = \frac{\sum_k N_k(\bar{x}_k - \bar{x})^2}{k-1} \tag{2}$$

and the within group variance as

$$s_{\text{within}} = \frac{\sum_k \sum_i (x_{ik} - \bar{x}_k)^2}{N-k}. \tag{3}$$

The denominators of the variances give the degrees of freedom of each.

The F-score is calculated with the following formula:

$$F = \frac{s_{\text{between}}}{s_{\text{within}}}. \tag{4}$$

The p-value can then be sampled from the F-distribution using the F-score and the two computed degrees of freedom.

Chi Squared

Chi-squared test uses the homonymous probability distribution as test statistic. This is a wide family of tests. Among the most used chi-squared tests we have *Pearson's Chi-Squared Test* and *Yates's Correction for Continuity*.

The first test is usually applied to sets of categorical data in order to assess with what likelihood observed differences among the empirical distributions may be due to chance. For example given N distributions divided among n classes, Pearson's test can be used to assess if the assumption that the classes have uniform probability is to be rejected or not. Indeed, in general, the empirical class frequency will be different from the theoretical frequency:

$$F_c = \frac{N}{n}. \tag{5}$$

However, this difference may be not sufficient to state that the distribution is not uniform. Pearson's test allows us to do so in a principled way by computing the value of the test statistics as:

$$\chi^2 = \sum_{i=1}^{n} \frac{(O_i - E_i)^2}{E_i} \tag{6}$$

$$= N \sum_{i=1}^{n} p_i \left(\frac{O_i/N - p_i}{p_i} \right)^2. \tag{7}$$

The p-value, and therefore an answer to whether the divergence from the uniform distribution is statistically significant, can then be easily assessed comparing such value to the the chi-squared distribution with $n - 1$ degrees of freedom.

Ensembling Selections

When several Feature Selection techniques show a good performance, we tried to find a consistent subset of features selected by them. This is done in a naive and conservative manner, by taking the intersection of the features selected across experiment repetitions and methods.
